# Bone marrow mesenchymal stromal cells support translation in refractory acute myeloid leukemia

**DOI:** 10.1016/j.celrep.2024.115151

**Published:** 2025-01

**Authors:** Livia E. Lisi-Vega, Alice Pievani, María García-Fernández, Dorian Forte, Tim L. Williams, Marta Serafini, Simón Méndez-Ferrer

**Affiliations:** 1 Cambridge Stem Cell Institute, Cambridge CB2 0AW, UK; 2 Department of Haematology, University of Cambridge, Cambridge CB2 0AW, UK; 3 National Health Service Blood and Transplant, Cambridge CB2 0AW, UK; 4 Tettamanti Center, Fondazione IRCCS San Gerardo Dei Tintori, 20900 Monza, Italy; 5 Department of Veterinary Medicine, University of Cambridge, Cambridge CB3 0ES, UK; 6 School of Medicine and Surgery, University of Milano-Bicocca, 20900 Monza, Italy; 7 Instituto de Biomedicina de Sevilla-IBiS (Hospitales Universitarios Virgen Del Rocío y Macarena/ CSIC/ Universidad de Sevilla), 41013 Seville, Spain; 8 Departamento de Fisiología Médica y Biofísica, Universidad de Sevilla, 41009 Seville, Spain

## Abstract

In acute myeloid leukemia (AML), malignant cells surviving chemotherapy rely on high mRNA translation and their microenvironmental metabolic support to drive relapse. However, the role of translational reprogramming in the niche is unclear. Here, we found that relapsing AML cells increase translation in their bone marrow (BM) niches, where BM mesenchymal stromal cells (BMSCs) become a source of eIF4A-cap-dependent translation machinery that is transferred to AML cells via extracellular vesicles (EVs) to meet their translational demands. In two independent models of highly chemo-resistant AML driven by MLL-AF9 or FLT3-ITD (internal tandem duplication) and nucleophosmin (NPMc) mutations, protein synthesis levels increase in refractory AML dependent on nestin+ BMSCs. Inhibiting cap-dependent translation in BMSCs abolishes their chemoprotective ability, while EVs from BMSCs carrying eIF4A boost AML cell translation and survival. Consequently, eIF4A inhibition synergizes with conventional chemotherapy. Together, these results suggest that AML cells rely on BMSCs to maintain an oncogenic translational program required for relapse.

## Graphical abstract

**Graphical abstract:**
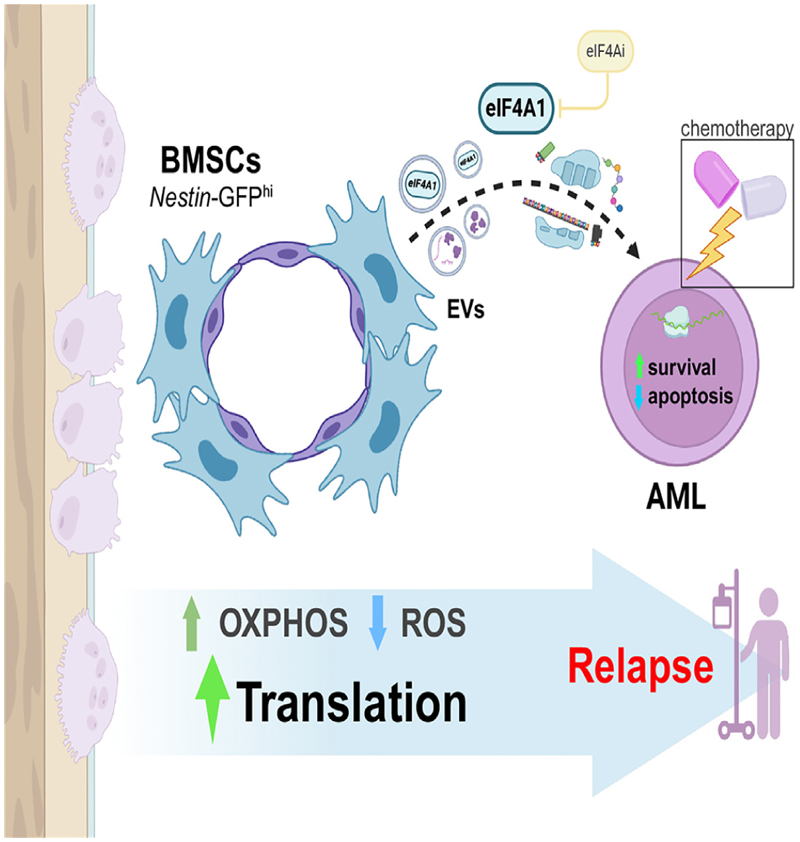

## Introduction

Acute myeloid leukemia (AML) impedes normal blood production and causes fatigue, infections, bleeding problems, and enlarged spleen and liver (due to infiltration by leukemic cells).^1^ More than 70% of pediatric AML cases carry a genetic alteration affecting a specific gene (MLL1), which is often associated with a poor outcome because the mutated cells of origin behave as stem cells (leukemia stem cells [LSCs]), which can survive current therapies and drive relapse.^2^ Mutations in the type III receptor tyrosine kinase FLT3 are present in ~30% AML cases, and combined mutations in FLT3 (FLT3-ITD [internal tandem duplication]) and nucleophosmin (NPMc) confer poor prognosis.^3^

During malignancy, the bone marrow (BM) microenvironment is modified to support and protect malignant clones through a variety of mechanisms, including neoangiogenesis, activation of survival pathways, protection from oxidative stress, immunosuppression, and, ultimately, promotion of therapeutic resistance.^4^ Among niche cells, BM mesenchymal stromal cells (BMSCs) transfer functional mitochondria to leukemic blasts upon chemotherapy, increasing mitochondrial ATP from the tricarboxylic acid (TCA) cycle and oxidative phosphorylation (OxPhos) and leading to a survival advantage in AML cells.^5–7^ Upon chemotherapy treatment, BMSCs transfer aspartate to AML blasts, fueling LSCs driving relapse.^8^ These data illustrate the importance of the metabolic crosstalk between BMSCs and leukemic blasts when exposed to selective pressures like chemotherapy.

Microenvironmental cells are exposed to the same stressors as tumor cells and similarly adjust their metabolism. Whereas previous studies have illustrated how translation regulation influences hematopoietic stem cell (HSC) differentiation and malignant transformation,^9–11^ our understanding of the niche’s translational reprogramming during leukemogenesis and relapse is very limited.^4^ A previous study has shown that genotoxic or infectious stress triggers tRNA transfer from osteoblasts to HSCs, modulating mRNA translation during stress hematopoiesis.^12^ In T cell acute lymphoblastic leukemia, tRNA deregulation allows for metabolic adaptation and leukemia growth.^13^ However, whether translational reprogramming in the niche maintains refractory AML is unknown and has been investigated here.

## Results

### Increased protein synthesis in AML cells and their niches supports chemo-resistance

**Figure 1:**
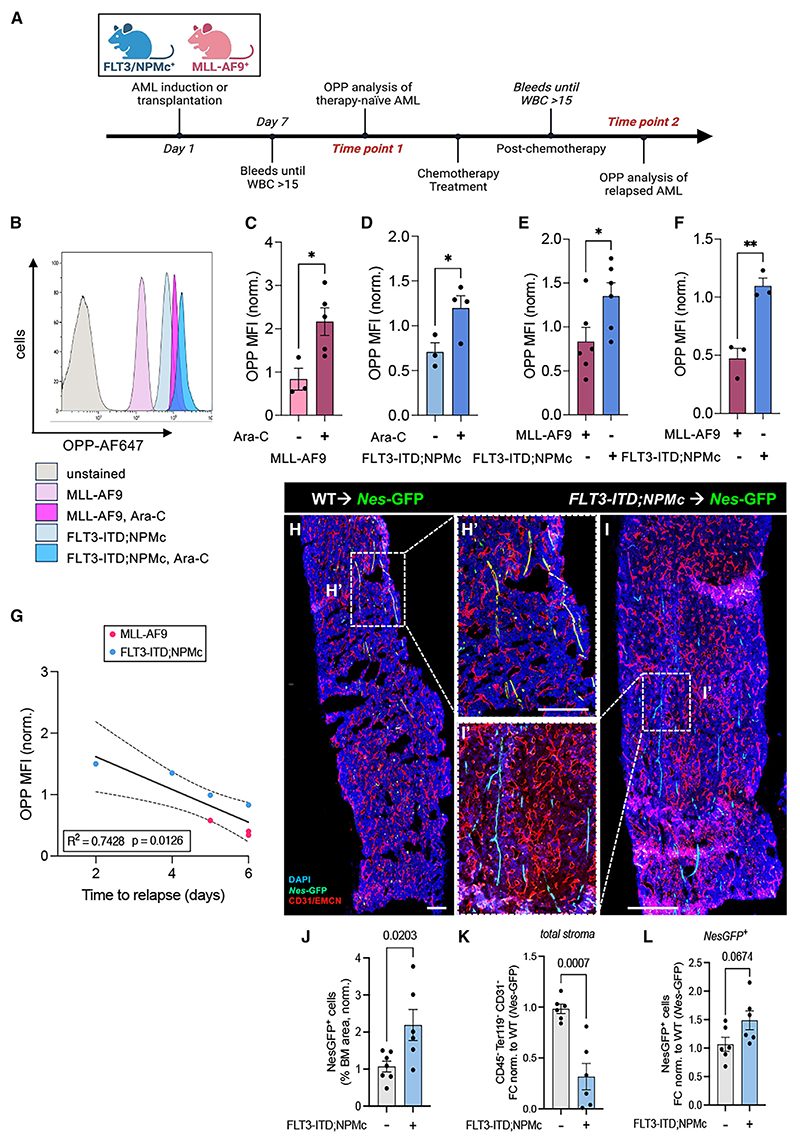
Increased protein synthesis in AML cells and their niches supports refractory AML (A) Experimental workflow of O-propargyl-puromycin (OPP) labeling *in vivo* to assess AML blast translation levels before and after AML recurrence in the FLT3-ITD; NPMc and MLL-AF9 mouse models. (B–D) Representative flow cytometry plots (B) and quantification (C and D) of global protein synthesis levels measured by OPP mean fluorescence intensity (MFI) in MLL-AF9 (C) or FLT3-ITD; NPMc (D) blasts from therapy-naive and refractory AML mice. MFI values are normalized to average OPP MFI values of therapy-naive AML mice. Dots represent data from individual mice (*n* = 2 independent experiments). Data are mean ± SEM. Unpaired two-tailed t test. (E and F) Comparison of global protein synthesis levels between MLL-AF9 and FLT3-ITD; NPMc AML blasts in the BM (E) and peripheral blood (F). Dots represent data from individual mice (data pooled from *n* = 4 independent experiments for E and *n* = 2 for F). Data are mean ± SEM. Unpaired two-tailed t test. (G) Inverse correlation between AML blast translation levels in refractory AML and the time to recurrence (measured as number of days post-chemotherapy until reappearance of disease based on peripheral blood counts). Dots represent data from individual mice. Data were pooled from MLL-AF9 (*n* = 3) and FLT3-ITD; NPMc (*n* = 4) *in vivo* experiments. Pearson correlation analysis and linear regression line fitting were used (blue line, 95% confidence interval [CI] represented by dashed line). (H and I) Representative BM immunofluorescence images of *Nestin-gfp* mice transplanted with WT (H) or FLT3-ITD; NPMc (I) BM cells. *Nestin*-GFP (green), CD31^+^ or endomucin (EMCN)^+^ blood vessels (red), and nuclei counterstained with 4′,6-diamidino-2-phenylindole (DAPI; blue). Scale bar, 200 μm. (J) BM area occupied by *Nes-*GFP^+^ cells (%) in *Nestin-gfp* mice 5–8 weeks after transplantation with WT or FLT3-ITD; NPMc AML cells. Areas are normalized with the average of WT controls from 2 independent experiments. Dots represent data from individual mice. Unpaired two-tailed t test. (K and L) Fold change in the number of BM stromal cells (CD45^−^Ter119^−^CD31^−^) (K) and BMSCs expressing *Nes*-GFP (Nes-GFP^+^) (L) in the BM of control (*Nes-GFP* mice) and AML (*Nes-GFP;FLT3-ITD;NPMc*) mice. Numbers were normalized with the average of WT controls. Dots represent data from individual mice. Unpaired two-tailed t test. See also Figures S1 and S2.

First, we measured mRNA translation levels *in vivo* in LSC-enriched cells isolated from two different mouse models of AML. In the *iMLL-AF9* model, mice^14^ express the MLL-AF9 oncofusion protein upon doxycycline-induced transactivation. Conversely, *FLT3-ITD;NPMc* mice were generated by intercrossing a knockin mouse carrying a human ITD within the juxtamembrane (JM) domain of exon 14 of the murine Flt3^15^ with a conditional knockin mouse model of the most common form of NPM1c mutation, type A.^16^ Both animal models develop AML manifested as increased white blood cell counts and splenomegaly (Figures S1A and S1B). Mice were administered O-propargyl-puromycin (OPP) to label newly synthesized proteins^9,17,18^ and analyzed after 1 h (Figure 1A). In both AML models, the OPP mean fluorescence intensity (MFI) was 2- to 3-fold higher in refractory LSCs compared to “therapy-naive” AML (Figures 1B–1D and S1C–S1F). In MLL-AF9 blasts, translation increased in response to chemotherapy treatment and preceded AML recurrence (Figures S1G and S1H). Global LSC translation was 2- to 3-fold higher in *FLT3-ITD;NPMc* mice than in *iMLL-AF9* mice and inversely correlated with time to AML recurrence (Figures 1E–1G). These results suggest that chemo-resistant AML cells strongly depend on their mRNA translation capacity to drive relapse.

Previous studies have suggested that AML relapse relies on the metabolic reprogramming of the microenvironment, involving cells such as adipocytes^19^ and BMSCs.^8^ We have previously shown that, compared with other stromal cells, HSC-niche-forming nestin^+^ BMSCs^20^ are preserved and promote AML bioenergetics and chemo-resistance in *iMLL-AF9* mice.^5^ To test whether nestin^+^ BMSCs are similarly spared in *FLT3-ITD;NPMc* AML, we measured *Nestin*-GFP^+^ cells in *Nes-*GFP mice^21^ transplanted with BM cells from *FLT3-ITD;NPMc* mice (Figures S2A–S2D). Resembling the results in *iMLL-AF9* mice,^5^ the number of BM CD45^−^Ter119^−^CD31^−^ cells was significantly reduced, whereas nestin^+^ BMSCs were preserved, or even expanded, in *FLT3-ITD;NPMc* AML (Figures 1H–1L), suggesting that nestin^+^ BMSCs might also contribute to leukemogenesis in this AML subtype.

### Chemotherapy induces a translational switch in nestin^+^ BMSCs

**Figure 2:**
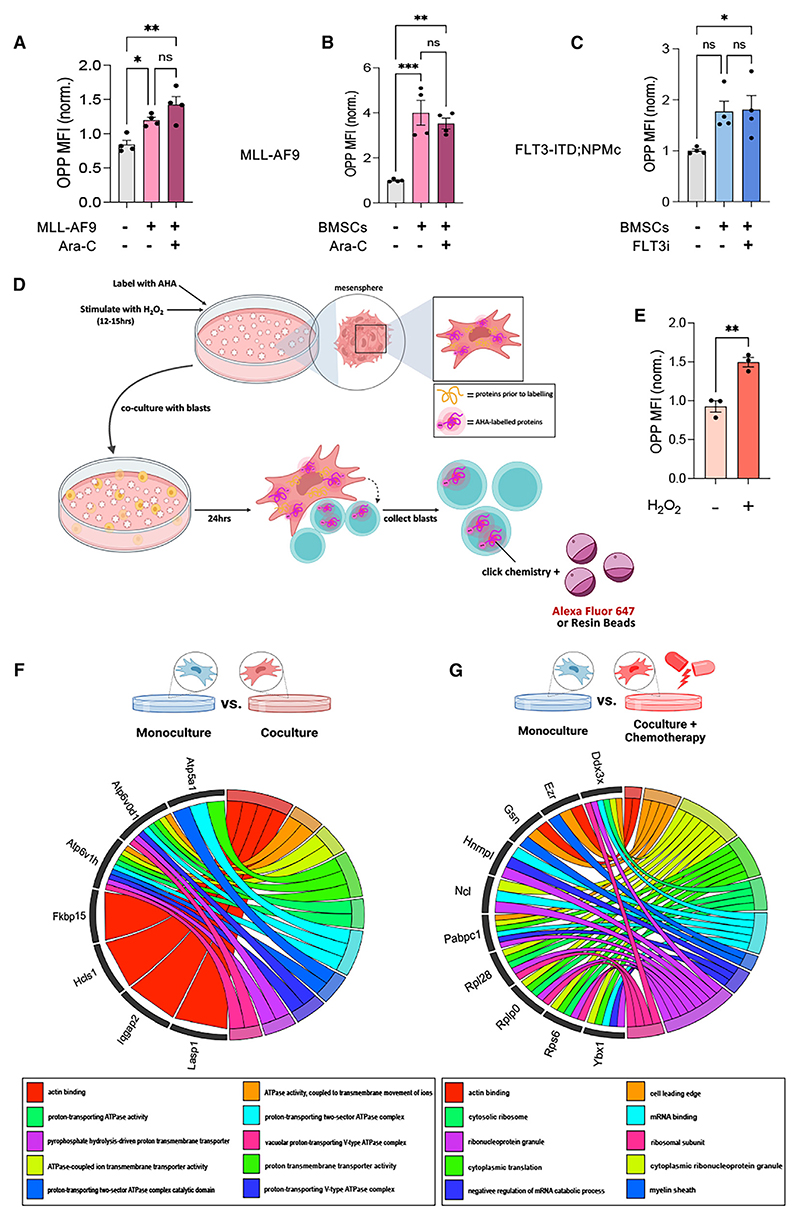
Chemotherapy induces a metabolic/translational switch in nestin+ BMSCs (A) *In vivo* global protein synthesis levels measured by O-propargyl-puromycin (OPP) mean fluorescence intensity (MFI) of CD45^−^CD31^−^Ter119^−^*Nes*-GFP^+^ cells from WT and AML mice before or after AML recurrence. (B and C) Global protein synthesis levels as measured by OPP MFI in monocultured BMSCs and BMSCs cocultured with MLL-AF9 (B) or FLT3-ITD; NPMc (C) AML blasts with/without chemotherapy (AraC or the FLT3i AC220, respectively, *n* = 4). Data are mean ± SEM. Dots represent biological replicates (*n* = 4 independent experiments). *p < 0.05, **p < 0.01, and ***p < 0.001. One-way ANOVA and pairwise comparisons. (D) Overview of BONCAT experiments. Briefly, AHA-labeled BMSCs previously stimulated with H_2_O_2_ (50 μM) were washed and cocultured with MLL-AF9 or FLT3-ITD; NPMc blasts for 24 h in the presence or absence of chemotherapy treatment. After coculture, AHA-labeled proteins were conjugated to a fluorophore (AF647) or resin beads via click chemistry for microscopy and proteomics, respectively. MS analysis aimed to (1) identify changes in the nascent proteome of BMSCs upon coculture and exposure to chemotherapy and (2) use the presence of the AHA label to trace proteins transferred from BMSCs to AML blasts. (E) Global protein synthesis levels as measured by OPP MFI in monocultured BMSCs and BMSCs previously stimulated with H_2_O_2_ (50 μM). **p < 0.01, unpaired two-tailed t test. (F and G) Chord diagrams showing the relationships between the top 10 Gene Ontology (GO) terms and their associated proteins, appearing as differentially translated/labeled in BMSCs in coculture vs. monoculture (F) and BMSCs cocultured in the presence of chemotherapy vs. monoculture (G). Top 10 GO terms were extracted from the GO enrichment results obtained from ClusterProfiler, and the associated genes were extracted from these terms. The top 10 genes were then extracted from this list and sorted based on their frequency of occurrence in the aforementioned GO terms. Proteomics samples pooled were together from various experiments, *n* = 2 for monoculture and *n* = 3 for coculture and coculture + chemotherapy conditions. See also Figures S1 and S2 and Table S2.

To get further insight, we conducted a supervised analysis of *Nes*-GFP^hi^ BMSC RNA sequencing (RNA-seq) data from AML mice (GEO: GSE140135),^5^ which showed an over-representation of categories related to translation (Table S1). Notably, *Nes*-GFP^hi^ BMSCs from AML patient-derived xenograft (PDX) models, including FLT3-ITD AML, similarly displayed increased protein synthesis in a previous study (GEO: GSE148625).^22^ This was validated in human AML: a supervised analysis of human BMSC RNA-seq data (GEO: GSE84881)^23^ revealed higher mRNA expression of ribosomal proteins in patients with AML from different genetic risk categories compared to healthy donors (Figure S2E), suggesting that BMSC translational rewiring in AML is conserved across species. Chemotherapy treatment was associated with increased translational proteins in the nascent proteome of BMSCs because a comparison of the nascent proteomes of BMSCs pre-treated with H_2_O_2_ and cocultured with AML blasts in the presence of chemotherapy vs. steady-state coculture yielded an enrichment of translation-related Gene Ontology (GO) categories and KEGG pathways in BMSCs cocultured with chemotherapy, in comparison to steady-state coculture (Figures S2F and S2G). Protein synthesis in BMSCs was higher in AML mice than in wild-type (WT) controls (Figure 2A); this was directly enforced by AML cells, as OPP MFI was 2- to 4-fold higher in BMSCs cocultured with AML cells compared to those that were monocultured (Figures 2B and 2C), suggesting that AML cells boost niche translation to meet their anabolic demands.

To investigate how AML shapes the translational profile of BMSCs, we performed biorthogonal non-canonical amino acid tagging (BONCAT) experiments.^24^ Nestin^+^ BMSCs were grown as mesenspheres, which exhibit increased *in vivo* self-renewal and support of HSCs compared with standard plastic-adherent BMSCs.^20,25,26^ Mesenspheres were incubated with the azide-bearing artificial amino acid L-Azidohomoalanine (AHA), which is incorporated into newly synthesized proteins instead of methionine,^24^ and treated with H_2_O_2_ to increase reactive oxygen species (ROSs), which are high in AML^27^ and a key driver of metabolic adaptation (Figure 2D). Indeed, H_2_O_2_ pre-treatment similarly increased translation levels in BMSCs (Figure 2E). Mass spectrometry (MS) analysis of the BMSC nascent proteome^28^ in coculture with AML blasts highlighted the increased production of ATP-synthesis-related proteins, while chemotherapy treatment increased the labeling of translation-related proteins (Figures 2F, 2G, S2F, and S2G; Table S2). These results suggest that chemotherapy treatment reprograms BMSC metabolism toward biosynthetic processes characterized by increased translation.

### Translation-related proteins are transferred from the BM niche to AML

**Figure 3:**
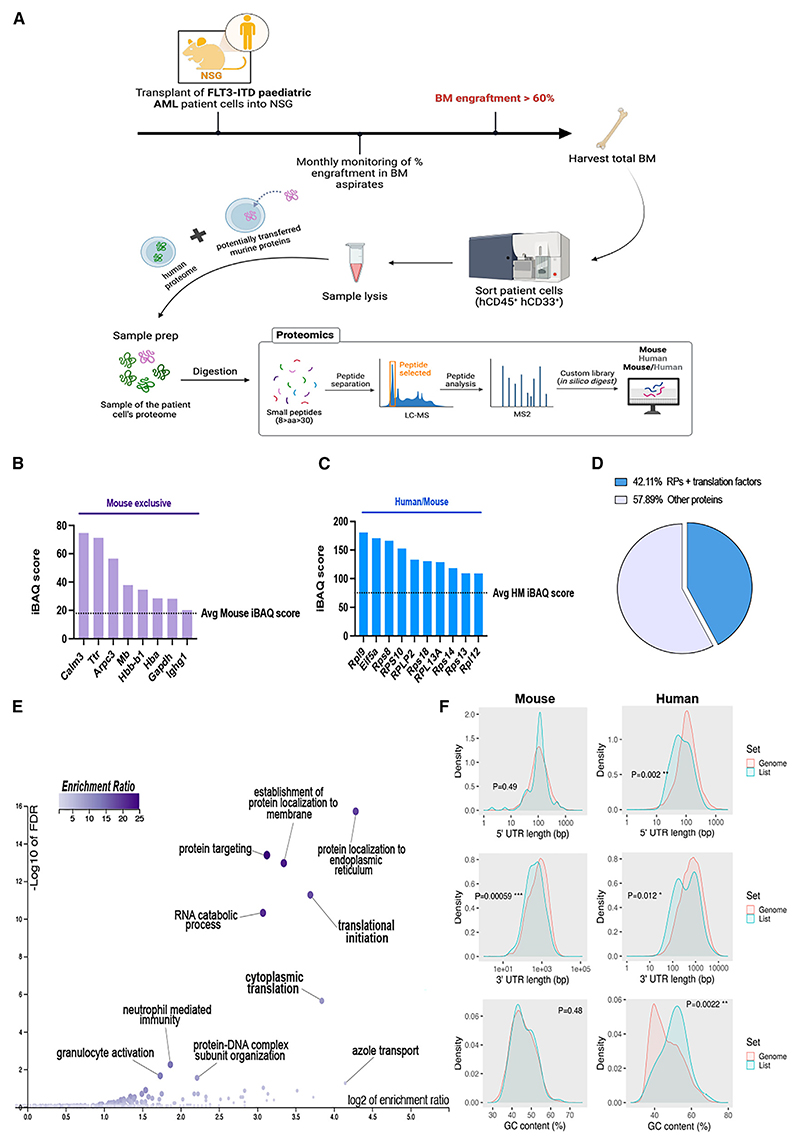
Translation-related proteins are transferred from the BM niche to AML blasts, supporting oncogenic translation (A) Experimental workflow to assess the potential transfer of microenvironmentally derived proteins to pediatric FLT3-ITD AML cells in a xenograft model. (B) iBAQ scores of mouse proteins enriched in xenografted hCD45^+^hCD33^+^FLT3-ITD AML cells (*n* = 3). The discontinuous line represents the average iBAQ score of the mouse proteins (M) identified in the samples. Proteins were considered enriched when the iBAQ observed/expected ratio + iBAQ scores were higher than average. (C) iBAQ scores of the top 10 translation related proteins enriched in xenografted hCD45^+^hCD33^+^FLT3-ITD AML cells (*n* = 3) mapped to human and mouse (HM) libraries. The discontinuous line represents the average iBAQ score of the HM mapped proteins identified in the samples. Proteins were considered enriched when the iBAQ observed/expected ratio + iBAQ scores were higher than average. (D) Frequencies (%) of ribosomal proteins (RPs) and translation factors found enriched in xenografted hCD45^+^hCD33^+^FLT3-ITD AML cells (*n* = 3). (E) Volcano plot of enriched Gene Ontology categories. (F) Distribution of 5′ and 3′ UTRs and GC content (%) in genes coding for mouse or human proteins enriched in in xenografted hCD45^+^hCD33^+^FLT3-ITD AML cells compared to other coding genes (ShinyGO software^33^). *p < 0.05, **p < 0.01, ***p < 0.001, and ****p < 0.0001. Unpaired two-tailed t test. See also Tables S3 and S4.

We next asked whether human (h) AML cells depend on their BM niches to augment their translational capacity at relapse. To that end, we generated a hAML PDX model. Human *FLT3-ITD* AML cells isolated from non-obese diabetic (NOD) severe combined immunodeficiency (SCID) gamma (NSG) mice with high (>60%) BM engraftment were analyzed by MS (Figure 3A). The resulting label-free spectra were compared against custom libraries of unique mouse and human tryptic peptides generated by *in silico* digest.^29,30^ 116 proteins from xenografted hAML blasts exclusively mapping to the mouse library included translation-related proteins (Tables S3 and S4). The intensity-based absolute quantification (iBAQ) values and iBAQ scores were obtained to calculate the absolute and relative protein abundances, respectively.^31^ iBAQ scores and observed/expected ratios higher than average marked 8/116 enriched mouse proteins and 49/1,912 conserved proteins (Figures 3B and 3C). Interestingly, 42% high iBAQ score proteins comprised ribosomal proteins and translation factors (Figure 3D). WebGestalt analysis of enriched proteins^32^ confirmed the over-representation of translation initiation and cytoplasmic translation GO categories (Figure 3E). Together, these results suggest the shuttling of translation-related proteins from the BM microenvironment to AML cells.

Cancer cells rely on cap-dependent translation of oncogenic mRNA transcripts.^34,35^ Pro-tumorigenic transcripts exhibit 5′ untranslated regions (UTRs) and high GC content, giving rise to mRNA secondary structures (such as G-quadruplexes) that require unwinding by the eIF4F complex helicase eIF4A.^36^ Analysis of mRNA transcripts corresponding to human-specific proteins enriched in xenografted AML cells revealed high GC content, which was not detected in transcripts encoding for mouse proteins derived from the host (Figure 3F, left). Human transcripts exhibited significantly shorter 5′ UTRs (Figure 3F, right), a feature commonly associated with highly translated transcripts in other cancers.^37,38^ Therefore, the microenvironmental supply of translation-related proteins might facilitate a “pro-oncogenic” translational program in AML cells.

### Nestin^+^ BMSCs support increased protein synthesis in refractory AML

**Figure 4:**
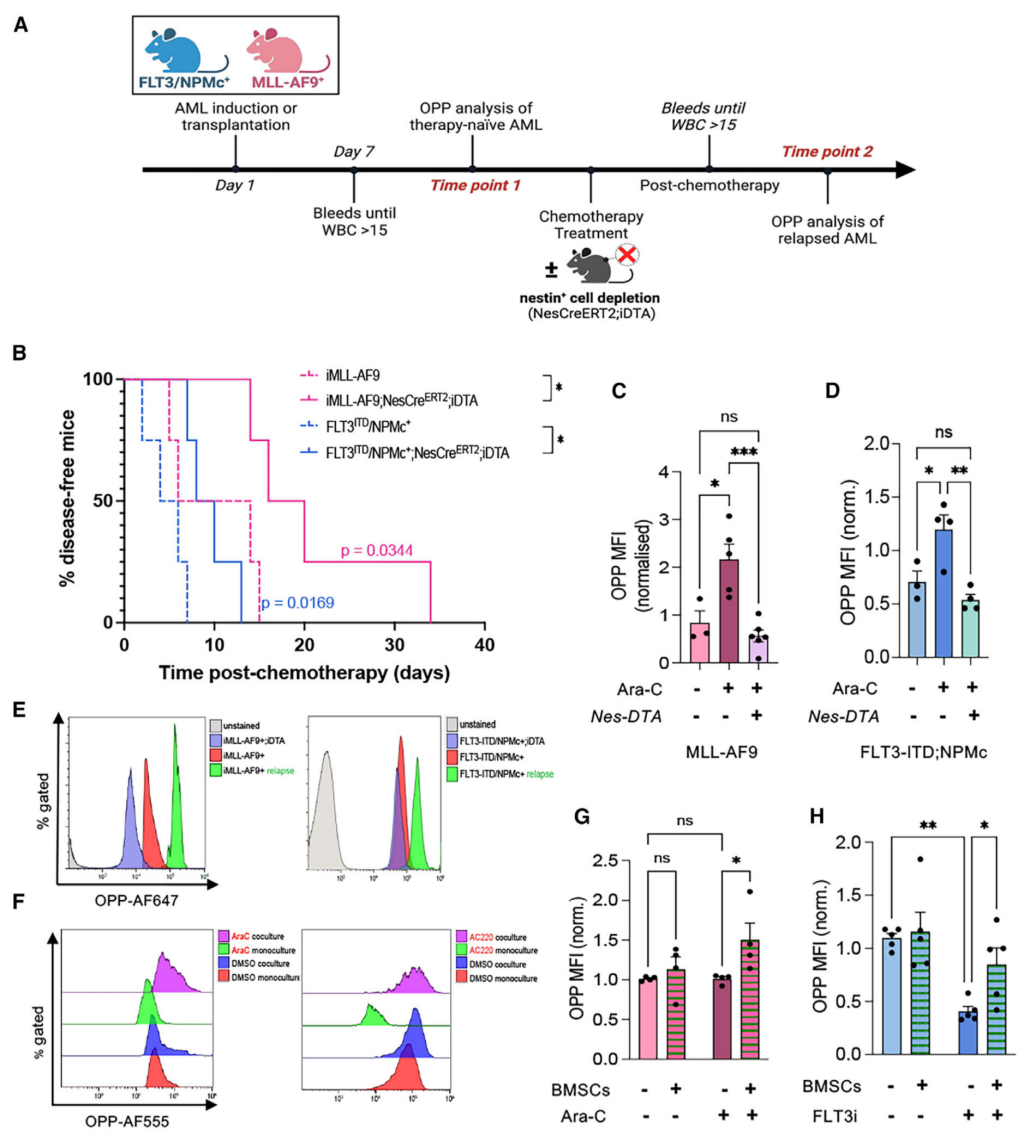
Nestin^+^ BMSCs support increased protein synthesis in refractory AML (A) Experimental workflow of *in vivo* O-propargyl-puromycin (OPP) labeling to assess blast translation levels at AML recurrence in control littermates or experimental mice with nestin^+^ cell depletion (*Nes-Cre*^*ERT2*^*;iDTA* mice). (B) Refractory AML in control iMLL-AF9 mice or FTL3-ITD; NPMc mice (dashed line, *n* = 4) compared with experimental AML mice following nestin^+^ cell depletion (*Nes-Cre*^*ERT2*^*;iDTA* mice; continuous lines, *n* = 4). *p < 0.05, log rank test. (C) Global translation in CD45.2^+^lin^−^ckit^+^MLL-AF9^+^ leukemia stem cells (LSCs) in therapy-naive mice (iMLL-AF9) vs. refractory AML mice with (*Nes-DTA*^+^) or without nestin^+^ cell depletion. (D) OPP mean fluorescence intensity (MFI) of Lin^−^CD45.2^+^ckit^+^FLT3-ITD; NPMc blasts from therapy-naive mice (FLT3^ITD^/NPMc^+^) vs. refractory AML mice with (*Nes-DTA*^+^) and without nestin^+^ cell depletion. (E) Global translation in CD45.2^+^lin^−^ckit^+^ LSCs from MLL-AF9 (left) or FLT3-ITD; NPMc (right) therapy-naive or refractory AML mice with (iDTA) or without nestin^+^ cell depletion. (F–H) Global translation flow cytometry plots (F) and quantification (G and H) in monocultured/cocultured MLL-AF9 (G) or FLT3-ITD; NPMc (H) AML blasts with/without chemotherapy. (C, D, G, and H) Each dot is a biological replicate (*n* = 4). Data are mean ± SEM. *p < 0.05, **p < 0.01, and ***p < 0.001; ANOVA and pairwise comparisons. See also Figures S2 and S3 and Tables S5 and S6.

To interrogate the possible function of enduring nestin^+^ BMSCs in AML translation *in vivo*, we used a mouse model that allows conditional depletion of nestin^+^ cells. Mice carrying tamoxifen-inducible Cre^ERT2^ recombinase under the control of *Nestin* regulatory elements (*Nes-Cre*^*ERT2*^)^39^ were intercrossed with the *R26lacZbpA*^*flow*^*DTA* strain,^40^ which carries a Cre-inducible diphtheria toxin A allele. To study the effects of nestin^+^ cell depletion in the two AML models, *Nes-cre*^*ERT2*^*;R26lacZbpA*^*flox*^*DTA* mice (abbreviated as *Nes-cre*^*ERT2*^;*iDTA*) were intercrossed with *iMLL-AF9* mice (*iMLL-AF9;Nes-cre*^*ERT2*^;*iDTA*) or transplanted with BM cells from *FLT3-ITD;NPMc* mice (*FLT3-ITD;NPMc; iDTA*), together with control helper cells. Upon AML development, *iMLL-AF9*;*Nes-Cre*^*ERT2*^;*iDTA* or *FLT3-ITD;NPMc;iDTA* mice and their control *iDTA* littermates were treated with chemotherapy alone, or in combination with tamoxifen, to deplete nestin^+^ cells (Figure 4A). In both AML models, nestin^+^ cell depletion at the time of chemotherapy delayed AML recurrence and reduced leukemic blasts in circulation or infiltrating the spleen (Figures 4B and S2H–S2K). Notably, nestin^+^ cell depletion prevented the increase of protein synthesis normally observed in refractory LSCs (Figures 4C and 4D), suggesting that nestin^+^ cells are required for optimal protein synthesis in refractory AML. This was not explained by the overall leukemic burden, as translation levels did not correlate with white blood cell counts or spleen weight and instead tended to be lower in AML mice with nestin^+^ cell depletion (Figures S3A and S3B). For confirmation, *FLT3-ITD;NPMc* or *iMLL-AF9* AML blasts treated with an FLT3 inhibitor (AC220) or Ara-C, respectively, were cultured alone or with BMSCs. Flow cytometry analysis confirmed that BMSCs increase global translation in both AML subtypes upon chemotherapy (Figures 4E–4H).

The mammalian target of rapamycin (mTOR) critically regulates protein synthesis in response to microenvironmental cues and nutrients through its effector proteins 4E-BP1 and S6K,^41^ which are two important regulators of cap-dependent translation.^42^ Consistent with the observed increase in global translation, coculture with BMSCs promoted 4E-BP1 and S6K phosphorylation in both AML subtypes upon chemotherapy treatment combined with serum starvation (Figures S3C–S3H). Furthermore, the increased translation observed in AML cells in coculture with BMSCs was accompanied by AML protection from cell death induced by the endoplasmic reticulum stressor thapsigargin in combination with chemotherapy treatment (Figures S3I and S3J). Together, these results highlight the dependency of chemo-resistant AML blasts on nestin^+^ BMSCs to meet their translational demands.

### Nestin^+^ BMSCs shape the nascent proteome of refractory AML

**Figure 5:**
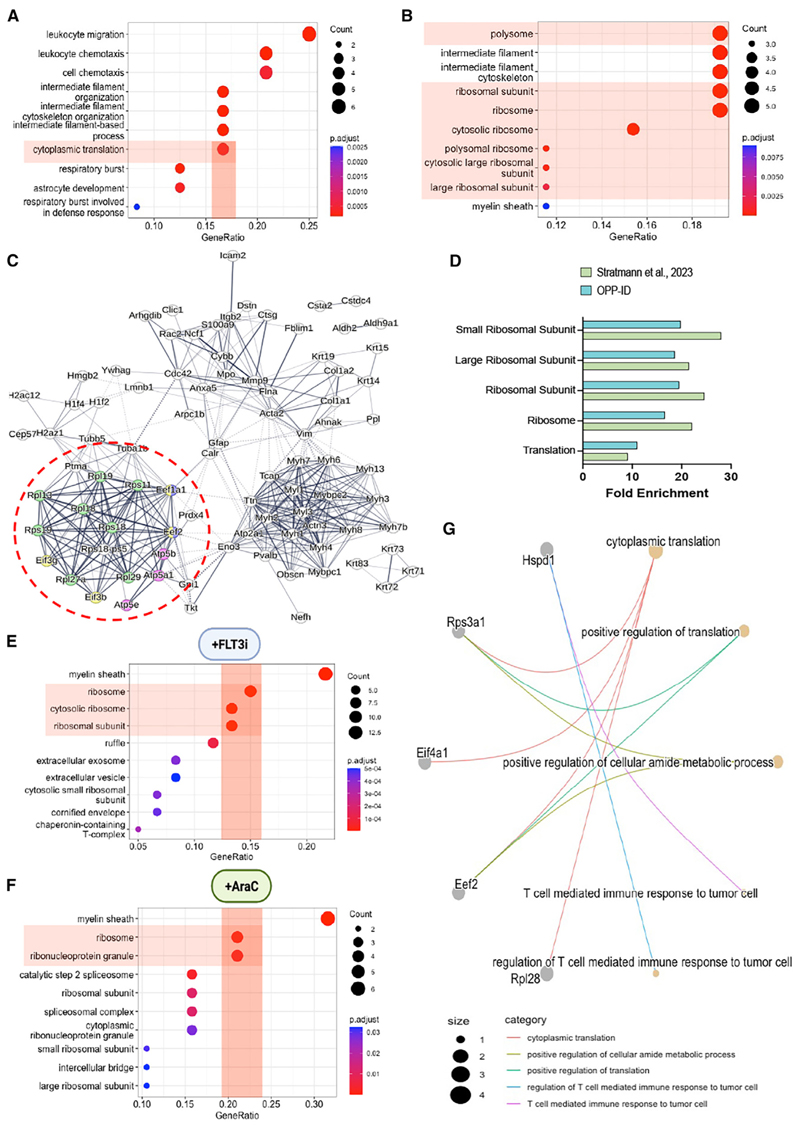
Nestin^+^ BMSCs shape the nascent proteome of refractory AML cells (A and B) Gene Ontology (GO) categories of biological process (A) and cellular component (B) terms enriched in nascent proteome of lin^−^Ly6G^+^CD11b^+^ cells from AML mice with (*iMLL-AF9;Nes-Cre*^*ERT2*^*;iDTA, n* = 9) or without (control *iMLL-AF9, n* = 8) nestin^+^ cell depletion. (C) Protein-protein interaction (STRING) analysis of differentially O-propargyl-puromycin (OPP)-labeled proteins in lin^−^Ly6G^+^CD11b^+^ cells from AML mice with (*iMLL-AF9;Nes-Cre*^*ERT2*^*;iDTA, n* = 9) or without (control *iMLL-AF9, n* = 8) nestin^+^ cell depletion. Interacting translation-related proteins are highlighted by the red discontinuous line. (D) Fold enrichment in translation-related GO categories enriched in AML mice and patients with relapsed AML.^46^ (E and F) GO cellular component categories enriched in proteins labeled with the azide-bearing artificial amino acid AHA and found to be transferred from BMSCs to cocultured FLT3-ITD; NPMc (E) or MLL-AF9 (F) AML blasts after chemotherapy with FLT3 inhibitor (FLT3i, AC220) or Ara-C, respectively. (G) CNet plot of transferred proteins shared by AraC-treated MLL-AF9 AML blasts and FLT3i-treated FLT3-ITD; NPMc AML blasts and their relationships to the top biological process GO terms enriched in both conditions (3 biological replicates from 2 independent experiments). See also Figure S4 and Table S5.

To investigate the translational dependency of AML blasts on nestin^+^ cells, we analyzed the nascent proteome of AML blasts isolated from OPP-injected *iMLL-AF9;Nes-cre*^*ERT2*^;*iDTA* mice or their control *iDTA* littermates (see Figure 4A). Newly synthesized proteins were covalently linked through OPP to resin beads via click chemistry and identified by MS.^43^ Comparison of the OPP-labeled proteomes of control and experimental mice revealed 93 differentially labeled proteins (Table S5). Interestingly, the AML blasts of mice with nestin^+^ cell depletion exhibited increased OPP labeling of proteins associated with GO categories related to protein translation and the ribosome (Figures 5A and 5B; Table S5). StringDB analysis and k-means clustering were used to evaluate differential protein-protein interaction networks.^44^ Consistent with GO analysis, 1/3 clusters comprised translation-related proteins, including translation factors (Eef2, Eif3g, Eef1a1) and ribosomal (Rps18, Rpl19, Rps11) and endoplasmic reticulum oxidative protein folding pathway (Prdx4) proteins^45^ (Figure 5C). The enrichment of translation-related categories observed in AML mice was shared with proteomic profiles from patients with relapsed AML ^46^ (Figure 5D), emphasizing translational changes as a hallmark of relapsed AML. Together with decreased protein synthesis in leukemic blasts from refractory AML mice following nestin^+^ cell depletion (see Figures 4C and 4D), the nascent proteome changes in AML blasts suggest a contribution from BMSCs to meet the translational demands of refractory AML.

### BMSCs transfer translation-related proteins to AML upon chemotherapy

BONCAT^24^ was used to trace proteins transferred from BMSCs to AML cells. BMSCs treated with H_2_O_2_ and labeled with AHA were cocultured with CellTracker Orange CMRA Dye-stained AML blasts, and AHA-labeled proteins from BMSCs were conjugated to Alexa Fluor 647 using click chemistry (see Figure 2D). Confocal analysis revealed CMRA^+^ AML blasts carrying the AHA-AF647 label corresponding to BMSC-derived proteins (Figures S4A and S4B).

To identify transferred proteins, CD45^+^ AML blasts were sorted and lysed, and AHA-labeled proteins were isolated through resin beads and analyzed by MS. GO analysis of the transferred proteins highlighted the enrichment of ribosome-related cellular component categories (Figures 5E and 5F; Table S6). This was not explained by the skewed labeling of ribosomal proteins, as only 6% of AHA-labeled proteins in monocultured BMSCs were ribosomal (Figure S4C). Notably, the transfer of translation-related proteins preferentially increased after chemotherapy (Figure S4D). Furthermore, BMSC-derived proteins were similarly enriched in GO terms associated with cytoplasmic translation or positive regulation of translation (Figure 5G). A cross-comparison of AHA labeling and xenograft experiments revealed the conservation of microenvironment (host-derived) translation-related proteins detected in hAML cells *in vivo*, which were also transferred from BMSCs to AML blasts in coculture (Figure S4E; Table S4). Altogether, these results suggest that translation-related proteins from BMSCs support a pro-oncogenic translational program in refractory AML.

### BMSCs support AML translation through eIF4A carried in EVs

**Figure 6:**
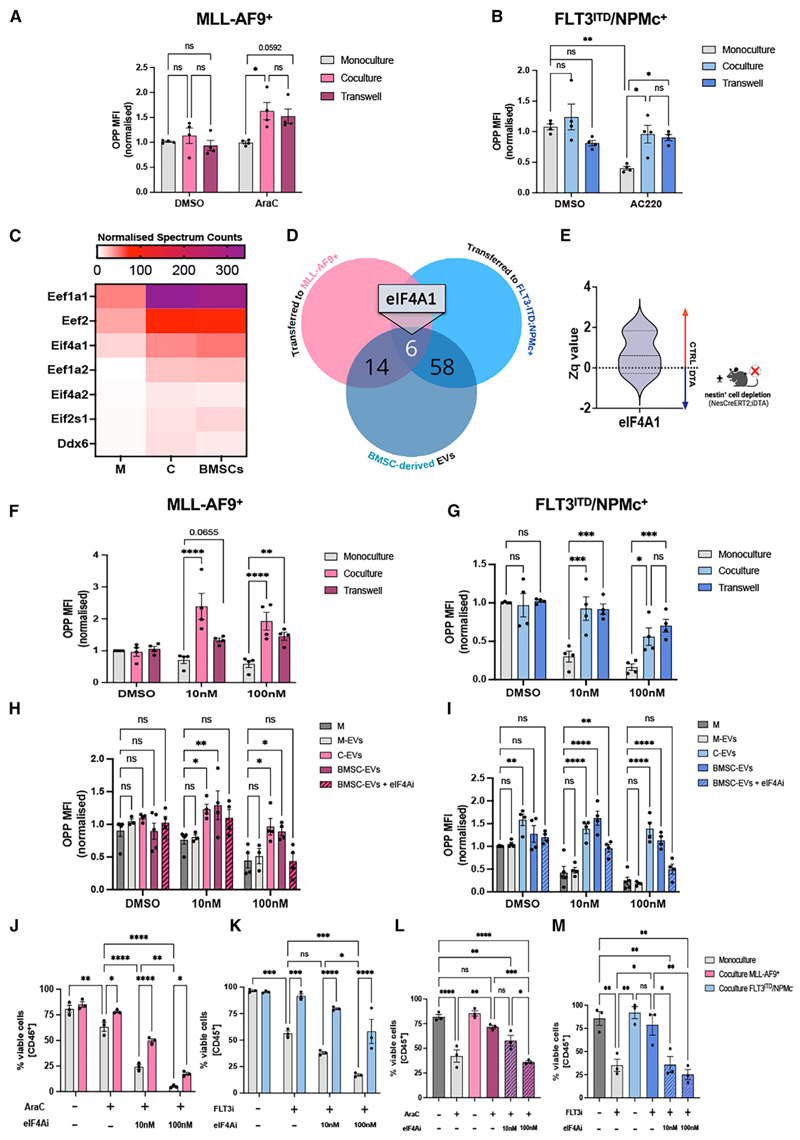
BMSCs support AML blast translation through eIF4A carried in extracellular vesicles (A and B) Global translation in MLL-AF9 (A) or FLT3-ITD; NPMc (B) AML blasts in monoculture or cocultured with BMSCs directly or through transwell, treated with chemotherapy (A, AraC; B, FLT3 inhibitor AC220) or control vehicle (DMSO) (*n* = 4). (C) Heatmap of average normalized spectrum counts of translation initiation and elongation factors detected in extracellular vesicles (EVs) derived from monocultured MLL-AF9 blasts (M), AML-BMSC cocultures (C), and BMSCs (*n* = 3). (D) Venn diagram of proteins transferred from BMSCs to MLL-AF9 blasts or FLT3-ITD; NPMc blasts and similarly detected in BMSC-derived EVs. eIF4A1 stands out among 6 shared proteins as a critical factor regulating pro-oncogenic translational programs. (E) Violin plot of average eIF4A1 Zq values in secretome of AML mice with (*iMLL-AF9;Nes-Cre*^*ERT2*^*;iDTA*) or without (control *iMLL-AF9*) nestin^+^ cell depletion (*n* = 3). (F and G) Global translation in MLL-AF9 (F) and FLT3-ITD; NPMc (G) blasts treated for 12 h with 10/100 nM eIF4A inhibitor (eIF4Ai) or control DMSO and maintained for 24 h in monoculture (M) or in direct (C) or transwell (T) coculture with BMSCs (*n* = 4). (H and I) Global translation in MLL-AF9 (H) and FLT3-ITD; NPMc (I) blasts pre-treated for 12 h with 10/100 nM eIF4Ai or control DMSO, followed by addition of EVs isolated from monocultured AML blasts (M-EVs), AML-BMSC cocultures (C-EVs), or monocultured BMSCs pre-treated with vehicle (BMSC-EVs) or 100 nM eIF4Ai (BMSC-EVs + eIF4Ai) for 12 h (*n* = 4). (J and K) Frequency of CD45^+^ MLL-AF9 (J) or FLT3-ITD; NPMc (K) AML cells resistant to 12 h treatment with eIF4Ai compared with control vehicle (DMSO), followed by monoculture or coculture with BMSCs in presence of chemotherapy (*n* = 3). (L and M) Frequency of surviving CD45^+^ MLL-AF9 (L) or FLT3-ITD; NPMc (M) AML cells treated with FLT3i and cultured alone or in coculture with BMSCs pre-treated for 12 h with eIF4Ai or control vehicle (*n* = 3). (B and F–M) Each dot is a biological replicate. Data are mean ± SEM. *p < 0.05, **p < 0.01, and ***p < 0.001; ANOVA and pairwise comparisons. See also Figure S5 and Tables S7 and S8.

Next, we investigated the mechanism of protein transfer. Global translation increased similarly in *iMLL-AF9* and *FLT3-ITD;NPMc* AML blasts cocultured with BMSCs directly or separated by 0.4 μm transwell (Figures 6A and 6B). Therefore, unlike mitochondrial transfer,^5–7,47^ the translational rewiring between BMSCs and AML cells does not require cell-cell contact.

Among contact-independent mechanisms, extracellular vesicles (EVs) are key players in AML-microenvironment intercellular communication.^48,49^ EVs transmit endoplasmic reticulum stress responses between AML cells and BMSCs^50^ and restrain normal HSC proliferation, conferring AML a competitive advantage.^51^ However, whether and how EVs regulate oncogenic translation in AML is unknown. EVs isolated from BMSCs and AML blasts showed high purity through transmission electron microscopy, nanoparticle tracking analysis, and MS identification of >90% of the top 100 EV protein markers from two well-established databases (93/100 for Vesiclepedia and 90/100 for ExoCarta) (Figures S4F–S4H and S5A; Table S7). Notably, translation factors (eEF2, eIF4A1, and eIF2s1) were markedly increased in EV fractions obtained from cocultures compared with AML cells alone (Figure 6C). Comparison with AHA experiments revealed a significant overlap (Figure 6D; Table S8). EVs contained 20 and 64 proteins similarly transferred from BMSCs to *iMLL-AF9* or *FLT3-ITD;NPMc* AML blasts, respectively. Among 6 proteins shared by all conditions, 4 were translation related (Rpl28, Rps3a, eEF2, and eIF4A1; Tables S6, S7, and S8). These results confirm our previous observations and underscore EVs as important mediators of AML-BMSC translational crosstalk.

eIF4A1 is the eIF4F complex helicase responsible for unwinding stable mRNA secondary structures to allow ribosome scanning and cap-dependent translation.^52^ eIF4A1 was 1 out of 6 proteins detected as transferred from BMSCs to AML blasts via EVs (see Figure 6D; Table S8) and increased in EVs from coculture, compared with AML monoculture (see Figure 6C). Furthermore, eIF4A1 appeared reduced in the AML BM secretome after nestin^+^ cell depletion (Figure 6E). These results advocated for eIF4A1 as a mediator of translational rewiring in the leukemic niche; therefore, we investigated its function in this process.

The marine natural product pateamine A (PatA) stabilizes eIF4A’s interaction with mRNA and prevents its release, inhibiting cap-dependent translation.^53^ The PatA analog des-methyl,des-amino PatA (DMDA-PatA; hereon eIF4Ai [eIF4A inhibitor]) displays potent anticancer activity.^54^ As expected, eIF4Ai pre-treatment inhibited translation and induced AML apoptosis (Figures S5B–S5E). However, cap-dependent translation in AML blasts pre-treated with eIF4Ai was 2- to 3-fold increased after coculture with BMSCs; these results were reproduced in transwell coculture and increased AML survival (Figures 6F, 6G, S5F, and S5G). Conditioned media from coculture or EVs isolated from BMSCs (but not AML cells) similarly rescued the translation in AML blasts pre-treated with eIF4Ai; most importantly, this translational boost was abrogated when pre-treating BMSC-derived EVs with eIF4Ai (Figures 6H, 6I, S5H, and S5I), confirming the role of EV-derived eIF4A in AML translation.

We evaluated the functional consequences of BMSC-dependent translational enhancement in chemo-resistant AML cells. In both AML models, eIF4Ai pre-treatment synergized with Ara-C or FLT3i, expanding to primary AML mouse models the enhanced the cytotoxic effects of eIF4Ai and chemotherapy previously observed in hAML cell lines^55^; yet, BMSCs increased AML cell survival after combination therapy (Figures 6J and 6K). Notably, inhibiting eIF4A in BMSCs before coculture did not affect their viability but rather suppressed their chemoprotective function (Figures 6L, 6M, S5H, and S5I), highlighting the need of BMSC translation for AML escape from chemotherapy. This effect was independent of ROS exchange since BMSCs decreased mitochondrial ROS levels in cocultured AML blasts regardless of eiF4Ai treatment (Figures S5J–S5M). Together, these results suggest that BMSC-derived EVs enhance proteo-synthesis in AML, favoring AML survival and regrowth.

### Nestin^+^ BMSCs boost AML translation *in vivo*

**Figure 7:**
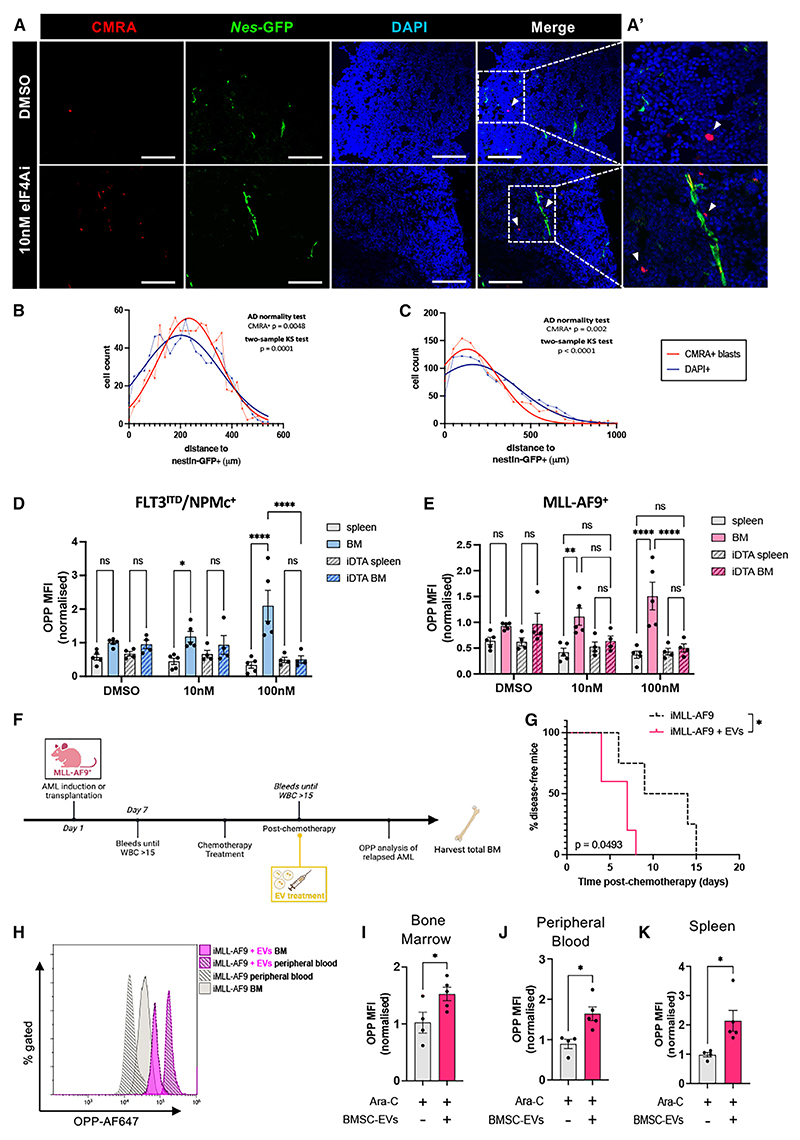
BMSCs rescue translation inhibition in AML cells *in vivo* (A and A′) Immunofluorescence of CellTrackerOrangeCMRA^+^ (red) AML cells, *Nestin*-GFP^+^ (green) cells and DAPI-counterstained nuclei (blue) in *Nestin-gfp* BM 12 h after homing of intravenously (i.v.) injected AML cells treated with eIF4A inhibitor (eIF4Ai) or vehicle (DMSO). Scale bar, 200 μm. (B and C) Cumulative frequency distribution representing the cartesian distances between i.v. injected CMRA^+^ blasts treated with control vehicle (B) or 10 nM eIF4Ai (C) and BM *Nes*-GFP^+^ cells compared with randomly distributed DAPI^+^ cells. Two-sample Kolmogorov-Smirnov and Anderson-Darling normality tests indicate the non-random distribution of AML cells, close to *Nes*-GFP^+^ cells (*n* = 3 mice per condition). (D and E) Global translation levels in CMRA^+^lin^−^CD45.2^+^ckit^+^ (D) FLT3-ITD; NPMc or (E) MLL-AF9 leukemic stem cells previously treated with vehicle or eIF4Ai and harvested for 12 h after i.v. transplantation from the BM or the spleen of recipient mice with (iDTA) or without nestin^+^ cell depletion. Dots represent biological replicates. (F) Scheme depicting experimental design of *in vivo* EV treatment experiments. (G) Kinetics of AML recurrence after chemotherapy treatment alone (black) or followed by i.v. injection of BMSC-derived extracellular vesicles (EVs, pink); *n* = 2 independent experiments, *p < 0.05, log rank test. (H–K) Flow cytometry histograms (H) and quantification of global translation in (I) BM, (J) peripheral blood, and (K) spleen lin^−^CD45^+^ckit^+^ cells from mice treated with chemotherapy alone (gray) or followed by BMSC-derived EV infusion (pink) (*n* = 2). (D–E and I–K). Data are mean ± SEM. (I–K) Each dot is a mouse. *p < 0.05, **p < 0.01, ***p < 0.001, and ****p < 0.0001. (D and E) Two-way ANOVA. (I–K) Unpaired two-tailed t test. See also Figure S6.

To further investigate the translational AML-BMSC crosstalk *in vivo*, CMRA-labeled AML blasts previously treated with eIF4Ai or vehicle were transplanted into *Nes-GFP* mice. Confocal BM analysis showed the non-random distribution of CMRA-labeled blasts close to *Nes*-GFP^+^ cells (Figures 7A–7C). In order to investigate the functional dependency *in vivo*, similar experiments were performed in mice with nestin^+^ cell depletion (Figure S6A). Normal host hematopoietic progenitor cells showed similar translation in the spleen and BM (Figure S6B). However, translation was significantly higher in the leukemic counterparts in BM compared with the spleen, dependent on the presence of nestin^+^ cells, as this effect disappeared upon nestin^+^ cell depletion (Figures 7D, 7E, and S6C). *In vivo*, treatment with BMSC-derived EVs in iMLL-AF9 mice accelerated AML recurrence (Figures 7F, 7G, S7D, and S7E), concomitantly with increased translation in AML blasts (Figures 7H–7K). These results provide evidence that BMSCs heighten proteo-synthesis in chemo-resistant AML cells *in vivo*.

## Discussion

Although current therapies effectively reduce the disease burden and induce remission, AML remains associated with a high relapse rate.^1^ Therefore, understanding AML recurrence is crucial for identifying vulnerability factors and eradicating AML. After chemotherapy, persisting AML cells acquire a distinctive metabolic state that favors treatment resistance.^8,56,57^ This process is facilitated by the microenvironment; for instance, BMSCs fuel AML metabolic adaptation and escape from chemotherapy.^5,8^

A key metabolic adjustment in AML is surged translation for building blocks. AML LSCs are highly dependent on amino acid metabolism and, through changes in their ribomethylome, redirect translation toward amino acid transporter mRNAs to increase intracellular amino acid levels.^58^ However, whether AML translation directly depends on BMSCs was not clear. One study has shown that mTOR is required for the BM stroma-dependent maintenance of protein translation and mitochondrial respiration in FLT3-inhibitor-resistant AML.^59^ We found that refractory AML cells increase proteo-synthesis in their BM niches, where BMSCs become a source of translational machinery that is transferred to AML cells via EVs, to meet their translational demands.

We used two independent models of highly chemo-resistant AML^2,3^ driven by MLL-AF9^14^ or FLT3-ITD; NPMc^15,16^ mutations. In both AML mouse models, AML proteo-synthesis increases when AML reappears and inversely correlates with the time to AML recurrence. Nestin^+^ BMSC depletion during chemotherapy alters the translational profile of AML and prevents increased proteo-synthesis, delaying AML recurrence.

PDX proteomics and *in vitro* AHA-labeling experiments revealed the transfer of translation-related proteins from the BM microenvironment—and specifically nestin^+^ BMSCs—to AML cells. Resembling mitochondrial transfer,^6,7,47^ BMSCs relay ribosomal proteins or translation-related factors to AML. But, as opposed to mitochondrial exchange, which mainly occurs through tunneling nanotubes,^6,7^ AML-BMSC translational crosstalk is mediated by EVs. Interestingly, stromal syntenin-1—a universal EV biomarker^60^—regulates AML survival and proteo-synthesis.^61^

AML (and cancer cells in general) rely on cap-dependent translation of oncogene mRNA transcripts,^34,35^ which exhibit mRNA secondary structures (such as G-quadruplexes) that require unwinding by the eIF4F complex helicase eIF4A.^36^ AML blasts overexpressing eIF4E become addicted to it, and treatment with ribavirin—a drug mimicking mRNA methylguanosine cap (m7G) and blocking eIF4E—inhibits tumor growth.^62,63^ In line with this, we found that xenografted hAML reflects a preference toward the cap-dependent translation of pro-tumorigenic transcripts (high GC content and short 5′ UTRs). In myelodys-plastic syndrome with high AML risk, malignant cells preferentially translate transcripts with short 5′ UTRs and high GC content through the binding of eIF4A to polyadenylate-binding protein 1 (PABPC1), leading to mRNA circularization, closedloop formation, and translation.^38^ We found high PABPC1 expression in BMSCs cocultured with chemotherapy-treated AML, while eIF4A1 increases in coculture-derived EVs (and decreases in the secretome of AML BM after nestin^+^ cell depletion). These findings led us to speculate that BMSCs support eIF4A-cap-dependent oncogenic translation in AML. We found that BMSCs rescue AML cap-dependent translation inhibition. Notably, EVs from BMSCs have the same effect and promote AML survival. Correspondingly, eIF4Ai treatment synergizes with conventional chemotherapy, eliminating AML cells. On the other hand, inhibiting cap-dependent translation in BMSCs abolishes their chemoprotective ability, highlighting the need for translational niche reprogramming in AML.

Chemo-resistant AML cells extensively benefit from increased OXPHOS and mitochondrial activity^64^ and rely on BMSCs for optimal bioenergetic and antioxidant capacity.^5^ Our results add an additional layer and suggest that AML cells also rely on BMSCs to maintain the high translational demand needed to survive chemotherapy and drive relapse, where a powerful regenerative response (to some extent mimicking stress hematopoiesis^65,66^) is needed to replenish the leukemic blast pool and drive AML resurgence.

### Limitations of the study

Most of the work presented here relies on murine models and would require validation in future studies that investigate different hAML subtypes and drug responses. While our study identifies mRNA translation and eIF4A as critical mediators of the translational rewiring in the refractory AML niche, it did not investigate the drivers of metabolic adaptation or the possible role of other cell types, which should be the focus of future research. Similarly, while our data suggest that chemotherapy treatment induces the adaptation of BMSCs to meet metabolic and translational requirements of AML cells, we cannot exclude the possibility that chemotherapy may also enhance EV uptake by AML cells or lead to other changes affecting AML translation.

## Resource Availability

### Lead contact

Further information and requests for resources and reagents should be addressed to the lead contact, Simon Mendez-Ferrer (sm2116@cam.ac.uk).

### Materials availability

New unique materials were not generated in this study.

## Star⋆Methods

Detailed methods are provided in the online version of this paper and include the following:

- Key Resources table
- Experimental Model and Study Participant Details
  ○ Patient samples
  ○ Mouse models
  ○ Bone marrow transplantation
- Method Details
  ○ Cell isolation and culture
  ○ Flow cytometry and fluorescent-activated cell sorting (FACS)
  ○ O-propargyl-puromycin (OPP) incorporation assay
  ○ mTOR phospho-flow
  ○ ROS detection and cell viability
  ○ Immunofluorescence
  ○ Extracellular vesicle (EV) isolation
  ○ Extracellular vesicle (EV) characterization
  ○ BONCAT proteomics
  ○ OPP-ID proteomics
  ○ PDX label-free proteomics
  ○ Protein identification
- Quantification and Statistical Analysis
  ○ EV and secretome proteomics data
  ○ Nascent proteomics data
  ○ Statistical analysis

## Star⋆Methods

### Key Resources Table

**Table:** 

REAGENT or RESOURCE	SOURCE	IDENTIFIER
Antibodies		
Biotin Mouse Lineage Panel (CD11b, Gr-1, Ter119, B220, CD3e)	BD Biosciences	559971 RRID:AB_10053179
Biotin Rat Anti-Mouse TER-119/Erythroid Cells (clone Ter119)	BD Biosciences	553672 RRID:AB_394985
Biotin Rat Anti-Mouse CD45 (clone 30-F11)	BD Biosciences	553078 RRID:AB_394608
Biotin Rat Anti-Mouse CD31 (clone MEC 13.3)	BD Biosciences	553371 RRID:AB_394817
Annexin-V FITC	BioLegend UK Ltd	640906
Alexa Fluor 647 Mouse anti-Ki-67	BD Biosciences	561126 RRID:AB_10611874
CD11 b PE (clone M1/70)	BioLegend UK Ltd	101208 RRID:AB_312791
CD45.1 APC (clone A20)	TONBO Biosciences	20-0453-U100
CD45.2 PerCP-Cy5.5 (clone 104)	BioLegend UK Ltd	109827 RRID: AB_893350
CD45.2 FITC (clone 104)	BioLegend UK Ltd	109805 RRID:AB_313442
ckit APC Cy7 (clone 2B8)	BioLegend UK Ltd	105856 RRID:AB_2876415
ckit FITC (clone 2B8)	BD Biosciences	561680 RRID:AB_10924598
Ly-6G/Ly-6C (Gr1) APC (clone HK1.4)	BioLegend UK Ltd	108412 RRID:AB_313376
Streptavidin BV711	BioLegend UK Ltd	405241
Streptavidin APC-Cy7	BioLegend UK Ltd	554063
Rat anti-CD31 (clone MEC13.3)	BD Biosciences	550274 RRID:AB_393571
Rat anti-endomucin (clone V.7C7)	Insight Biotechnology	sc-65495 RRID:AB_2100037
Rabbit Phospho-4E-BP1 (Thr37/46) (236B4)	Cell Signaling Technology	#2855 RRID:AB560835
Rabbit Phospho-S6 Ribosomal Protein (Ser235/236) (D57.2.2E)	Cell Signaling Technology	#4858 RRID:AB_916156
Rabbit mAb IgG XP® Isotype Control	Cell Signaling Technology	#3900 RRID:AB_1550038
Alexa Fluor 488 donkey anti-rabbit	Thermo Fisher Scientific	A21206 RRID:AB_2535792
Alexa Flour 647 donkey anti-rat	Abcam	ab150155 RRID:AB_2813835
Alexa Flour 647 donkey anti-goat	Thermo Fisher Scientific	A21447 RRID:AB_2535864
Chemicals, peptides, and recombinant proteins		
Fetal Bovine Serum (FBS)	NA	N/A
Fetal Bovine Serum (FBS), charcoal stripped	GIBCO	12676029
DMEM, high glucose, GlutaMAX™ Supplement, pyruvate	Thermo Fisher Scientific	31966021
RPMI Medium 1640 (1X) no phenol red	Thermo Fisher Scientific	11835–063
X-VIVO 20 Serum-free Hematopoietic Cell Medium	Lonza	LZBE04-448Q
Penicillin-Streptomycin	Thermo Fisher Scientific	15140122
Human IL-6	PeproTech	200-06-50
Murine IL-3	PeproTech	213-13-100
Murine SCF	PeproTech	216-16-50
Human PDGF-AA	PeproTech	100-13A-100
Human Oncostatin M (227 a.a.)	PeproTech	300-10T
Human FGF-basic (154 a.a.)	PeproTech	100-18B
Human EGF	PeproTech	AF-100-15
Human IGF-I	PeproTech	100–11
B-27 Supplement (50X), serum free-10 mL	Thermo Fisher Scientific	Cat. No. 17504-044
*N*-2 Supplement (100X)-5 mL	Thermo Fisher Scientific	Cat. No. 17502048
Trypsin (0.25%), phenol red	Sigma-Aldrich	Cat. No. T4049
Mercaptosuccinic acid	Sigma-Aldrich	M6182
Chicken Embryo Extract	Pajtler et al.^67^	N/A
Collagenase Type I (0.25%)	Stem Cell Technologies	#07902
BD IMag™ Streptavidin Particles Plus	BD Biosciences	557812
Red Blood Cell Lysis Buffer	BioLegend UK Ltd	420301
TNB (0.1 M Tris–HCl, pH7.5, 0.15 M NaCl, 0.5% blocking reagent)	Perkin Elmer	FP1020
Triton X-100	Sigma-Aldrich	T8787
DAPI	Thermo Fisher Scientific	D1306
DTT (dithiothreitol)	Thermo Fisher Scientific	R0861
Iodoacetamide	Sigma-Aldrich	I6125
Acetonitrile	Sigma-Aldrich	271004
Bovine Serum Albumin	Sigma-Aldrich	A7906
Cytarabine	Cayman Chemical	CAY16069
Quizartinib (AC220)	MedChem Express	HY-13001
DMDAPatA	Kuznetsov et al.^54^	N/A
Tamoxifen	Sigma-Aldrich	T5648
DMSO	Sigma-Aldrich	D5879
Doxycycline Hyclate = 98% (HPLC)	Sigma-Aldrich	D9891
Poly(2-hydroxyethyl methacrylate)	Sigma-Aldrich	P3932
DAKO Fluorescence mounting medium	Agilent Technologies	S3023
MitoSOX™ Red Mitochondrial Superoxide Indicator	Thermo Fisher Scientific	M36008
Monochlorobimane (mBCI)	Invitrogen	M1381MP
Corning™ Cell-Tak Cell and Tissue Adhesive	Thermo Fisher Scientific	CB40240
O-propargyl-puromycin (OPP)	Click Chemistry Tools	1407
L-Azidohomoalanine (AHA)	Click Chemistry Tools	1066
AZDye 555 Azide Plus	Click Chemistry Tools	1479
CellTracker™ Orange CMRA Dye	Thermo Fisher Scientific	C34551
Critical commercial assays		
Fixation/Permeabilization Solution Kit	BD Biosciences	554714
Pierce™ BCA Protein Assay Kit	Thermo Fisher Scientific	23225
Seahorse XF Real-Time ATP Rate Assay Kit	Agilent	103592–100
Click-&-Go Plus OPP (555 & 647) Protein Synthesis Assay Kit	Click Chemistry Tools	1494/1496
Click-&-Go™ Cell Reaction Buffer Kit	Click Chemistry Tools	1263
Click-&-Go Plus Protein Enrichment Kit *for capture of alkyne-modified proteins*	Click Chemistry Tools	1235
Click-&-Go Protein Enrichment Kit *for capture of azide-modified proteins*	Click Chemistry Tools	1033
Deposited data		
Mass spectrometry proteomics data (AHA and OPP-ID)	ProteomeXchange	PXD058646
Mass spectrometry proteomics data from PDX mice	ProteomeXchange	PXD058648
Experimental models: Organisms/strains		
congenic CD45.1 C57BL/6 mice	Charles River Laboratories	JAX:002014
congenic CD45.2 C57BL/6 mice	Charles River Laboratories	JAX:000664
NOD/SCID/IL2rg^−/−^ (NSG) mice	Charles River Laboratories	JAX:005557
*NesCre* ^*ERT2*^	Balordi et al.^39^	N/A
*Nestin-gfp* mice	Mignone et al.^21^	N/A
*rtTA;MLL-AF9* (iMLL-AF9) mice	Stavropoulou et al.^14^	N/A
*R26lacZbpA*^*flow*^*DTA* mice	Brockschnieder et al.^40^	N/A
*FLT3-ITD* mice	Lee et al.^15^	N/A
*Npm1*^*flox-cA/+*^ mice	Vassiliou et al.^16^	N/A
Software and algorithms		
ImageJ Software	Java	N/A
GraphPad Prism v9	GraphPad	N/A
FACS Diva Software	BD Biosciences	N/A
Kaluza Analysis Software	Beckman	N/A
Microsoft Excel	Microsoft Office	N/A
Zen 3.0 (blue edition)	Zeiss	N/A
LASX Software	Leica	N/A
Scaffold Software	Proteome Software	N/A
MaxQuant	Max Planck Institute of Biochemistry	N/A
Rstudio	Posit	N/A
DEP package	Zhang et al.^68^	N/A
Biorender.com	Biorender	N/A
Illustrator	Adobe	N/A

### Experimental Model And Study Participant Details

#### Patient samples

Experiments were conducted in accordance with the ethical standards of the Declaration of Helsinki, and with Italian national and international guidelines. AML sample collection, banking, and use were conducted according to Fondazione IRCCS San Gerardo dei Tintori institutional review board protocols. Written informed consent was obtained from patients’ legal representatives.

Bone marrow (BM) or peripheral blood (PB) samples were obtained from residual diagnostic material of three pediatric AML patients. Mononuclear cells were isolated using Ficoll-Paque Plus (GE Healthcare) and frozen in freezing media until the use.

#### Mouse models

8-12 week-old male or female (age and sex-matched) mice were used for experiments. Since no correlation was found between sex and nestin^+^ niches, both male and female mice were used in this study. *iMLL-AF9* mice,^14^ *FLT3-ITD;NPMc* mice,^15,16^ *Nes-gfp* mice,^21^ *Nes-Cre*^*ERT2*^ mice,^39^ *R26lacZbpA*^*flow*^*DTA* strain,^40^ NOD SCID gamma (NSG) mice, CD45.2 or CD45.1 C57BL/6J mice (Charles River Laboratories) were used in this study.

Mice were housed in specific pathogen free facilities in individually ventilated cages under 12h light/darkness cycles and controlled temperature (19–23)ºC and humidity (55 ± 10%) with free access to standard rodent chow (LabDiet 5021-3). Mice were housed in IVC cages, all diet was irradiated and cages/bedding/environmental enrichment was autoclaved. Full cage changes were performed in changing stations and any procedures are carried out in a CLII cabinet. The Health Monitoring Surveillance Program consisted of the microbiology analysis of mouse sentinels and contact animals following the FELASA recommendations. Every quarterly period, sentinels and contact animals of the rack were bled for serology and tested for the agents recommended by FELASA (http://www.felasa.eu/recommendations/recommendation/recommendations-for-health-monitoring-of-rodent-and-rabbit-colonies/). FELASA PLUS screening was performed annually and Klebsiella spp. was analyzed as an additional agent. All screenings revealed no significant findings. All animal procedures conformed to the United Kingdom Home Office regulations (PPL 70/8406 and P0242B783), Italian Ministry of Health, EU Directive 2010/63EU, Recommendation 2007/526/EC and Italian national low (D.L. n. 26/2014) regarding the protection of animals used for experimental and other scientific purposes, and were approved by the local ethics committees and the Animal Protection Area of Cambridge (UK) and the University of Milano-Bicocca.

#### Bone marrow transplantation

In order to follow the development of normal and leukemic hematopoiesis simultaneously in the same animals, we competitively transplanted lethally irradiated recipients (12 Gy whole body irradiation, split dose 6.0 + 6.0 Gy, 3h apart) with non-induced (CD45.2) 10^6^ BM cells from *iMLL-AF9* mice, or 2.5 x 10^5^ BM cells from *FLT3-ITD;NPMc* mice, together with 10^6^ BM cells from CD45.1 B6 mice. *iMLL-AF9* mice were kept with doxycycline (pellet food 400 mg/kg) from 2 weeks after transplant to induce transgene expression. Across the whole duration of the study, disease progression was monitored weekly through tail peripheral blood samples which were analyzed using an automated blood counter. Leukemia manifested in mice upon reaching 15x10^3^/mm^3^ peripheral white blood counts (WBC), with increased frequencies of monocytes and granulocytes. At this point, mice were treated or sacrificed. BM, spleen and blood were analyzed by cell counts, histology and flow cytometry, and cells were used for functional studies as required.

Different manipulations of the BM microenvironment were achieved by using diverse recipient strains. In order to selectively deplete Nes+ cells, *Nes-cre*^*ERT2*^ mice^39^ were crossed with a mouse line harboring a Cre-inducible diphtheria toxin gene (iDTA),^40^ yielding *Nes-cre*^*ERT2*^;R26lacZbpA^flox^DTA mice (abbreviated as *Nes-DTA*). Cre^ERT2^ recombinase was activated by tamoxifen treatment (Sigma). For combined Nes+ cell depletion and chemotherapy administration, *Nes-DTA* and control mice were simultaneously treated with tamoxifen (140 mg/kg, i.p., 3 doses on alternate days) and Ara-C (5 daily doses of 100 mg/kg, i.p.), after becoming leukemic (WBCs >15x10^3^/mm^3^). For adoptive transfer experiments, WT, *Nestin-gfp* and *Nes-cre*^*ERT2*^;*iDTA* mice were divided into 3 recipient groups, each receiving 10^6^ CMRA-labelled leukemic blasts (labeled with 5μM CellTracker Orange CMRA for 20mins in the dark at 37°C) previously treated *in vitro* with either vehicle (DMSO) or 2 different eIF4Ai (10nM and 100nM DMDAPatA) doses. To analyze protein synthesis *in vivo*, OPP was administered via i.p. injection to the mice as previously described (Click Chemistry Tools, 49.5mg OPP/kg, pH 6.4–6.6 in PBS).^18^ Vehicle solution (PBS) was administered to control mice. Mice were sacrificed at the appropriate time points and tissues were collected for flow cytometry and histology analysis.

To explore how BMSC-derived extracellular vesicles (EVs) influence AML translation *in vivo*, control and experimental mice were treated with either BMSC-derived EVs or PBS following chemotherapy (Ara-C). More specifically, mice were given intravenous injections of either 200μL of PBS or an EV suspension, corresponding to a final dose of 10^11^ EV/injection. Injections were carried out daily after chemotherapy treatment until the disease recurrence (WBCs >15x10^3^/mm^3^).

To generate primary AML PDX mice, male NOD/SCID/ILIIrg^−/−^ (NSG) mice (age 6 to 7 weeks) were purchased from Charles River Laboratories and intravenously injected with 5–10 million mononuclear cells obtained from AML patient. Femoral bone marrow aspirations were performed 8 weeks post-AML injection and every 8 weeks thereafter to monitor engraftment. AML engraftment percentages were determined by FACS analysis (FACSCanto II, BD). When signs of overt leukemia appeared, mice were sacrificed and the long bones were flushed in 5 mL of cold PBS/1%FBS. For sorting, BM cells were stained using the same three-color panel used to monitor engraftment, resuspended in PBS, filtered through a 70 μm sieve, and sorted using a BD FACSAria I instrument. Cell doublets and clumps were removed with electronic doublets discrimination gating. Post-sorting analysis showed purity >95%. Sorted cells were processed for proteomics analysis.

## Method Details

### Cell isolation and culture

Mesenspheres were cultured from mouse primary BM cells in the following way. Clean mouse bones were crushed in a mortar with 2 mL of a solution containing Collagenase Type I (0.25%) (Stem Cell Technologies). The suspension was incubated for 45 min at 37°C in agitation. After addition of PBS+ 2% FBS and passage through a 40 μm cell strainer, erythrocytes were lysed by incubation on ice with RBC Lysis Buffer. After this, erythroid, endothelial and hematopoietic cells were removed by magnetic depletion after incubation with biotin-conjugated primary antibodies against CD45, Ter119 and CD31 (BD Biosciences, 1:100) and subsequent incubation with streptavidin-conjugated magnetic beads (BD Biosciences). For sphere formation, the cells immunomagnetically depleted of hematopoietic (CD45^+^), erythroid (Ter119+) and endothelial (CD31^+^) cells were plated at low density (<500,000 cells/cm^2^) in ultralow-adherence 35 mm dishes (StemCell Technologies) after treatment with Poly-Hema (Sigma). The growth medium for spheres contained 0.1 mM β-mercaptoethanol; 1% nonessential amino acids (Sigma); 1% N2 and 2% B27 supplements (Invitrogen); recombinant human fibroblast growth factor (FGF)-basic, recombinant human epidermal growth factor (EGF), recombinant human platelet-derived growth factor (PDGF-AA), recombinant human oncostatin M (227 aa OSM, 20 ng/mL) and recombinant human IGF-1 (40 ng/mL; Peprotech) in Dulbecco’s modified Eagle’s medium (DMEM)/F12 (1:1)/human endothelial (1:2) serum-free medium (Invitrogen). Mesensphere medium was supplemented with 15% CEE prepared as described previously^67^ with minor modifications. Briefly, fertilized chicken eggs were incubated for 11 days at 38°C in a humidified incubator. Embryos were washed with DMEM (Invitrogen), macerated by passage through a 50 mL syringe and diluted 1:1 in the same medium. Hyaluronidase (2 mg, Sigma) was added to each 50 mL tube and incubated 1h at 4°C with agitation. Following 6 h ultracentrifugation (3 ×10^4^ *g*) at 4°C was decanted and filtered with 0.45 μm and 0.22 μm sterile filters (Millipore). Aliquots were stored at –80°C until use. The cultures were incubated at 37°C with 5% CO_2_, 20% O_2_ in a water-jacketed incubator and left untouched for 1 week. Afterward, half-medium changes were performed twice a week. For passage, spheres were enzymatically dissociated with 100μL Trypsin (EDTA-free) for 10 min at 37°C, applying mechanical dispersion every 10 min. The cells were washed with PBS once and replated with mesensphere medium in ultralow-adherence 35mm dishes (StemCell Technologies).

MLL-AF9 and FLT3-ITD; NPMc mouse leukemic blasts were isolated from bones of iMLL-AF9 and *FLT3-ITD;NPMc* mice. MLL-AF9 blasts were maintained in 6 well plates in RPMI 1640 (Invitrogen) without phenol red and supplemented with 10% charcoal-stripped FBS (Gibco), recombinant murine IL3, recombinant murine SCF and recombinant human IL-6 (10 ng/mL) (Peprotech), 1% Penicillin-Streptomycin, 1 μg/ml doxycycline. FLT3-ITD; NPMc blasts were maintained in 6 well plates in XVIVO20 medium (Lonza) supplemented with 5% FBS, 1% Penicillin-Streptomycin, recombinant murine IL3, recombinant human IL-6 (10 ng/ml) and recombinant murine SCF (50 ng/ml) (Peprotech). Cells were kept at 37°C in a water-jacketed incubator with 5% CO_2_ and 20% O_2_ and split every other day. MLL-AF9 were seeded at 500,000 cells/ml and FLT3-ITD; NPMc at 50,000 cells/ml.

We set up co-cultures systems with mesenspheres (~200 per mL) and leukemic blasts (250,000 cells/ml) for 24h. For MLL-AF9 blasts, cocultures were kept in RPMI1640 without phenol red and 10% charcoal-stripped FBS (Gibco) ± cytarabine (AraC, Cayman Chemical, 1μM). For FLT3-ITD; NPMc blasts, cocultures were kept in XVIVO20 and 5% FBS ± quizartinib (AC220, MedChemExpress, 1μM). All cocultures were set up in flat-bottom 96-well or 24-well low adherence tissue cultures plates (Costar) at 37°C in a water-jacketed incubator with 5% CO_2_ and 20% O_2_. Cultures were grown for 24h before flow cytometry staining (apoptosis, ROS levels, protein synthesis levels) or proteomics analysis.

Transwell cultures were set up in 24-well non-treated TC plates with transwell inserts of 0.4um pore size (VWR, 734–2742) with mesenspheres (~200 per mL) and leukemic blasts (250,000 cells/ml) for 24h. Transwell cultures were kept in RPMI1640 ± cytarabine and XVIVO20 ± quizartinib as outlined above for MLL-AF9 and FLT3-ITD; NPMc blasts respectively.

### Flow cytometry and fluorescent-activated cell sorting (FACS)

Briefly, after co-culture experiments, cell suspension with both types of cells (leukemic blasts and mesenspheres) was centrifuged for 5 min at 300 x g and resuspended in appropriate volume (~1mL). Then, non-aggregated single cells (most of leukemic blasts) were filtered out by passing through Test Tube with Cell Strainer Snap Cap (35μm nylon mesh) (Corning Falcon) in order to separate aggregated mesenspheres from leukemic blasts. After flipping the strainer onto a new tube to release all aggregate and after visually confirming that the aggregates have been rinsed off the filter, leukemic blasts (passed through the filter cap) were collected and washed. Leukemic blasts were stained with specific markers (namely CD45) in order to gate positive leukemic blasts (mainly CD45^+^) from negative stromal fraction for FACS experiments.

Leukemic blasts (monocultured or cocultured with BMSCs) were incubated with the appropriate dilution (2–5 μg/mL) of fluorescent antibody conjugates and were stained in PBS containing 2% FBS at 4°C. After incubation, cells were washed and stained with 4′,6-diamidino-2-phenylindole (DAPI, 1:2000) for dead cell exclusion or to confirm successful membrane permeabilization (depending on the experiment). Samples were analyzed using a Gallios flow cytometer (Beckman Coulter, Miami Lakes, FL) and Kaluza software (Beckman Coulter) or sorted using a BD FACSAria cell sorter equipped with FACSDiva software (BD Biosciences).

For immunophenotyping of hematopoietic cell populations, bones (limbs) were crushed in a mortar and spleen was cut in small pieces and tissue was disrupted mechanically in PBS with a syringe plunger. The resulting cell suspensions were filtered through a 40-μm mesh and depleted of red blood cells by lysis in 0.15 M NH_4_Cl for 10 min at 4°C. Blood samples were directly lysed in the same buffer for 10 min at room temperature. Cells (1-2 x 10^6^ cells/sample) were incubated with fluorescent antibody conjugates and DAPI as outlined above and analyzed with a Gallios flow cytometer (Beckman Coulter) and Kaluza software. The following antibody conjugates were used: CD45.1-APC (A20), CD45.2-FITC (104), CD45.2-PerCP-Cy5.5 (104), c-kitAPC Cy7 (2B8) or ckit-FITC (2B8), CD11b-PE (M1/70), Ly6G-APC (HK1.4) (all from BioLegend UK except CD45.1-APC from Insight Biotechnology), and biotinylated lineage antibodies from BD BioSciences (CD11b, Gr-1, Ter119, B220, CD3ε). Biotinylated antibodies were detected with APC-Cy7 or BV711-conjugated streptavidin (BioLegend UK).

To monitor PDX engraftment, a FACS antibody panel consisting of mCD45-PE (clone 30-F11, eBiosciences, used at 1:3500 dilution), hCD45-PerCP (clone 2D1, BD BioSciences, used at 1:100 dilution), and hCD33-PECy7 (clone P67.6, BD BioSciences, used at 1:33 dilution) was used.

### O-propargyl-puromycin (OPP) incorporation assay

Global protein synthesis levels were measured *in vitro* and *in vivo* using the OPP incorporation assay. *In vitro*, cells were incubated for 45 min in the corresponding culture medium containing 20μM of OPP. *In vivo*, OPP was administered as described above. After OPP labeling, cell suspensions were prepared and immunostained as described. Afterward, cells were fixed using the Cytofix/Cytoperm fixation/permeabilization kit (BD Biosciences, 554714). For the ClickIT reaction, either the Click-&-Go Plus OPP Protein Synthesis Assay Kit and azide conjugated to Alexa Fluor 555 or Alexa Fluor 647 (Click Chemistry Tools) were used to perform the azide-alkyne cycloaddition. Cells were incubated in the Click Chemistry mix in dark for 30 min at room temperature, washed twice with 1x BD Perm-Wash buffer and stained with DAPI. OPP fluorescence was measured using flow cytometry. OPP signals were normalized to whole BM controls or control wells after subtracting the autofluorescence background.

### mTOR phospho-flow

Leukemic blasts (monocultured or cocultured with BMSCs) were incubated with the appropriate dilution (2–5 μg/mL) of fluorescent antibody conjugates and were stained in PBS containing 2% FBS at 4°C. After staining, AML blasts were fixed using the Cytofix/Cytoperm fixation/permeabilization kit (BD Biosciences, 554714) for 20mins in the dark at 4°C. Fixed cells were then washed twice with 1x BD Perm-Wash buffer. Intracellular staining of p-S6K and p-4EBP1 was performed by incubating the cells with 100μL anti-p-S6K, anti-p-4EBP1 antibody or rabbit mAb IgG isotype control (1:150) diluted in 1x BD Perm-Wash buffer in the dark for 30 mins at RT. After incubation with primary antibody, cells were washed twice with 1x BD Perm-Wash buffer and incubated with 100μL AF488 conjugated secondary antibody (anti-Rabbit IgG (H + L) AF488, 1:200) diluted in 1x BD Perm-Wash buffer in the dark for 30 mins at RT. Lastly, cells were washed twice with 1x BD Perm-Wash buffer and resuspended in 200μL 1x BD Perm-Wash buffer supplemented with DAPI (1:2000).

### ROS detection and cell viability

Mitochondrial reactive oxygen species (ROS) were detected by staining cells with MitoSOX Red Mitochondrial Superoxide Indicator (Thermo Fisher) following manufacturer’s recommendations. In addition, the cell-permeant probe monochloromobimane (mBCI, Molecular Probes) was used for quantifying glutathione levels inside cells.

For the determination of apoptotic cells, samples were washed with PBS after surface antibody staining (if required) and subsequently stained with Annexin V-FITC (1:130 in PBS +2% FBS) for 10 min in the dark at 4°C and then supplemented with 100μL DAPI (1:2000 final concentration in PBS +2% FBS) before analysis.

### Immunofluorescence

#### In vitro

AML cells were previously labeled with 5μM CellTracker Orange CMRA for 20mins in the dark at 37°C, then washed and cocultured as outlined above. After fixation, permeabilization and blocking, cells were washed once with 3% BSA in PBS. For the click chemistry AHA labeling experiments, cells were then incubated with 250μL of click chemistry reaction cocktail (Click-&-Go Plus 647 imaging kit, CCT-1320) for 25-30 mins at RT and then washed with 3% BSA in PBS. If needed, cells were then incubated with primary and then secondary antibodies diluted 1:200 in 5% BSA for 1hr and 30 mins at RT respectively. Lastly, cells were counterstained with 5 mM DAPI in PBS for 10 mins at RT, washed with PBS and then imaged with a confocal microscope (Zeiss980) using 20× and 40× objectives. The acquired images were analyzed with ImageJ.

#### In vivo

Immunofluorescence staining of long bone cryosections was performed as described previously,^69^ with minor modifications. Briefly, cryosections of 10μm were thawed at RT for 15mins and rinsed with PBS. Sections were then incubated with 0.05% Triton X-100 (Sigma) in Tris-NaCl-blocking (TNB) buffer (0.1 M Tris-HCl, pH 7.5, 0.15 M NaCl, 0.5% blocking reagent, PerkinElmer) at RT, for 1 h. When needed, samples were incubated overnight at 4°C with conjugated antibodies: anti-EMCN (1:100, sc-65495, Insight Biotechnology) and anti-CD31 (1:100, catalog no. 550274, BD Biosciences) diluted in TNB +0,05% Triton X-100. Samples were washed 3 times with PBS +0,05% Triton X-100 for 5mins and incubated at RT for 1hr with secondary antibody: donkey-α-goat AF647 (catalog no. A21447, Thermo Fisher Scientific) and donkey-α-rat AF647 (1:300, catalog no. ab150155, Abcam) diluted in TNB. Samples were then washed another 3 times with PBS +0,05% Triton X-100 for 5mins and 1 time with PBS for 5 min. Finally, stained tissue sections were counterstained for 10mins with 5 mM DAPI in PBS and rinsed with PBS. For sections, slides were mounted in mounting medium (catalog no. S3023, DAKO). Images were acquired with a confocal microscope (Stellaris) using 10× and 20× objectives and analyzed with ImageJ and R studio.

### Extracellular vesicle (EV) isolation

Extracellular vesicles (EVs) used in experiments were isolated through 2 different methods to exclude any potential bias related to the isolation procedure.

#### Ultracentrifugation

Supernatant was collected from monocultures or co-cultures plated at a concentration of 2x10^5^ AML cells/well in 1mL of medium in a 24-well plate. Several wells from the same condition were pooled together to reach 5mL total volume. Supernatants were centrifuged at 300g for 10min, transferred to new tubes and centrifuged again at 2000g for 10mins to remove any remaining large cell debris. The supernatant obtained was then filtered with a 0.22μm PES filter and subjected to ultracentrifugation at 100,000g for 70 min at 4°C using an Optima XPN-80 ultracentrifuge with SW 40 Ti swinging rotor (Beckman Coulter, Brea, California, USA). Pellets were resuspended in PBS and frozen in 50-100μL aliquots at –80°C for later use and characterization.

#### Polymer-based (PEG) isolation

For polymer-based EV isolation, the ExoQuick-TC kit was used (Cat no. EXOTC10A-1, System Biosciences, Mountain View, California). Briefly, supernatants were collected from monocultures and co-cultures and centrifuged at 3000g for 15min to remove cells and cell debris. Supernatants were transferred to a new sterile vessel and incubated with the appropriate volume of ExoQuick TC (1mL of ExoQuick TC per 5mL of biofluid) for at least 12 h at 4°C (following manufacturer’s instructions). After incubation, the mixture was centrifuged at 1500g for 30 mins at room temperature to pellet EVs. The resulting EV pellet was centrifuged again at 1500g for 5mins to remove any residual ExoQuick-TC solution. EV pellet was resuspended in the appropriate volume of tissue culture media or PBS for use *in vitro* assays or further characterization.

#### EV isolation and eIF4Ai treatment

Media was collected from BMSC monocultures, centrifuged at 300g for 10min, transferred to new tubes and centrifuged again at 2000g for 10mins to remove any remaining large cell debris. eIF4Ai was then added to the remaining supernatant to a final concentration of 100nM and incubated at 4°C overnight. After incubation, EVs were obtained by polymer-based isolation using the ExoQuick TC kit as described above and used in our *in vitro* experiments.

### Extracellular vesicle (EV) characterization

#### Nanoparticle tracking analysis (NTA)

EVs used in *in vitro* and *in vivo* experiments were diluted 1:1000 or 1:5000 (depending on sample concentration) with PBS in order to obtain a concentration between 5 x 10^7^ -9 x 10^8^ particles/ml for NTA analysis using Nanosight NS500 (Malvern Instruments). 0.22μm-filtered PBS was used as a negative control. Samples were injected into the chamber at a constant flow rate using a syringe pump system and at least three 60s videos were recorded per sample. Data was analyzed using the NTA 3.2 software at a detection threshold between 5 and 6.

#### Transmission electron microscopy (TEM)

Glow-discharged copper-carbon film grids (400 mesh; EM Resolutions, Sheffield, United Kingdom and Quorum K100X glow discharger) were placed on top of a 5μL droplet of sample (on dental wax) for 2 min. After incubation, grids were transferred to a fresh drop of distilled water for 5s to remove buffer salts. Excess fluid was then removed with filter paper and the grids transferred to one drop of uranyl acetate (1.4% in distilled water) and incubated for 1min. Excess fluid was removed again with filter paper and the grids air dried prior to imaging.

Samples were imaged using a Tecnai G2 transmission electron microscope run at 200keV accelerating voltage with 20μm objective aperture to improve contrast. Image acquisition was carried out with an ORCA HR high resolution CCD camera using a Hamamatsu DCAM board running the Image Capture Engine, from AMT Corp. (Advanced Microscopy Techniques Corp. Danvers, USA).

#### EV proteomics sample preparation

EVs from different conditions including blasts cultured alone (*n* = 3), blast-spheres co-cultures (*n* = 3), spheres cultured alone (*n* = 3) were obtained. Each sample was generated by pooling cells from different cultures due to the scarcity of material. EVs samples were lysed with 50 mM Tris-HCl pH 7.5; 2% SDS, 10 mM TCEP (Tris(2-carboxyethyl) phosphine hydrochloride (TCEP) and centrifuged at 15,000 rpm for 15 min. Protein concentration was determined using a Direct Detect IR spectrometer (Millipore).

### BONCAT proteomics

Mesenspheres were collected, washed once with PBS and resuspended in the appropriate volume of fresh, prewarmed RPMI1640. Mesenspheres were then incubated for 30 min to allow for the depletion of methionine, which competes with AHA for incorporation into nascent polypeptide chains. Following methionine depletion, mesenspheres were labeled with AHA (4mM final concentration) and if applicable, treated with H_2_O_2_ to a final concentration of 50μM. Mesenspheres were incubated with AHA for 24hrs before coculturing.

24h later, the AHA-labelled mesenspheres were collected and washed twice with PBS before coculturing. Cocultures were set up with the appropriate leukemic blast to mesensphere ratio and treated with Ara-C or AC220 (1μM). Cocultures and their appropriate controls were grown for 24hrs prior to harvesting and proteomics sample preparation. After incubation, cultures were harvested and filtered to separate leukemic blasts from mesenspheres. The leukemic blasts passed through the filter were then immunostained for CD45 and DAPI, and alive blasts (CD45^+^, DAPI-) obtained by fluorescent-activated cell sorting (FACS). The sorted leukemic blasts were then lysed (Urea Lysis Buffer: 8M urea, 200mM Tris pH 8, 4% CHAPS, 1M NaCl supplemented with protease and phosphatase inhibitors) and then AHA-labelled proteins captured using a Click-IT protein enrichment kit (Invitrogen) according to the manufacturer’s standard protocol.

### OPP-ID proteomics

OPP-tagged cell populations of interest were sorted from the total BM samples from experimental and control mice. Unlabeled mouse BM was used as a negative control. The Click & Go Protein Enrichment kit (Click Chemistry Tools, #1235) was used to capture OPP-tagged proteins on resin beads following manufacturer’s instructions. Briefly, samples were lysed on ice for 15-30min with 500μL of lysis buffer containing protease inhibitors. Lysates were then centrifuged at 10,000g for 5mins and placed on ice. At this point, a BCA assay was performed to determine the protein concentration of each sample to normalize the quantity of protein used in the click chemistry reaction. The corresponding volume of each sample was mixed with the 2x copper catalyst solution and alkyne agarose resin and rotated end-over-end on a sample rotator for 16-20h. After incubation, the lysate/agarose resin click reaction was centrifuged and washed with 18 MΩ water. The agarose resin-bound proteins were reduced and alkylated steps with 1M DTT and 40mM iodoacetamide respectively. After this, the agarose resin was transferred to a spin column and subjected to several washing steps with agarose wash buffer with SDS, followed by washes with 8 M urea/100 mM Tris pH 8 and lastly, 20% acetonitrile. 500μL of digestion buffer were then added to the resin and the mixture was transferred to a labeled Eppendorf tube. The resin was then pelleted by centrifugation and handed over to the Cambridge Center for Proteomics (CCP) for trypsin digestion and MS analysis.

### PDX label-free proteomics

Sorted human AML blasts (hCD45^+^, hCD33^+^) were lysed in RIPA buffer (Thermo Fisher, 89900) supplemented with protease inhibitor cocktail (1:100, Sigma P8340) and Phenylmethanesulphonyl fluoride (PMSF) protease inhibitor (1:100, Sigma 93482). Lysates were run on 1D SDS-PAGE gels. 4 Individual gel bands were then excised from the whole gel lane and in-gel digestion of the proteins performed for each band. Briefly, the gel pieces were cut into 2mm square pieces, destained and subjected to reduction and alkylation (DTT and iodoacetamide) and digested overnight with trypsin at pH 8°C and 37°C. The peptide samples were run on a Thermo Orbitrap Q Exactive Plus Mass Spectrometer and spectra processed in MaxQuant.

### Protein identification

#### EV and secretome protein identification

Labeled peptides were analyzed by LC-MS/MS using a C-18 reversed phase nano-column (75 μm I.D. x 50 cm, 2 μm particle size, Acclaim PepMap RSLC, 100 C18; Thermo Fisher Scientific) in a continuous acetonitrile gradient consisting of 0–30% B in 360 min, 50–90% B in 3 min (A = 0.1% formic acid; B = 90% acetonitrile, 0.1% formic acid). A flow rate of 200 nL/min was used to elute peptides from the nano-column to an emitter nanospray needle for real time ionization and peptide fragmentation on an Orbitrap Fusion mass spectrometer (Thermo Fisher). An enhanced FT-resolution spectrum (resolution = 70,000) followed by the MS/MS spectra from the Nth most intense parent ions were analyzed along the chromatographic run. Dynamic exclusion was set at 40s.

For peptide identification, all spectra were analyzed with Proteome Discoverer (version 2.1.0.81, Thermo Fisher Scientific) using SEQUEST-HT (Thermo Fisher Scientific). For database searching at the Uniprot database containing all sequences from mouse and contaminants (April 27, 2016; 48,644 entries), the parameters were selected as follows: trypsin digestion with 2 maximum missed cleavage sites, precursor and fragment mass tolerances of 2 Da and 0.02 Da, respectively. Carbamidomethyl cysteine (+57.021 Da) and TMT modifications (+229.162932 Da) at N-terminal and Lys residues were selected as fixed modifications, and methionine oxidation (+15.994915 Da) as dynamic modification.

##### BONCAT and OPP-ID protein identification

Peak lists obtained from MS/MS spectra were analyzed using Mascot (Matrix Science, London, UK; version 2.7.0). Mascot was set up to search the cRAP_20190401.fasta; CCP_UniProt_Mus_musculus_20190621 database (unknown version, 63550 entries) assuming the digestion enzyme trypsin. Mascot was searched with a fragment ion mass tolerance of 0.100 Da and a parent ion tolerance of 20 PPM. Carbamidomethyl of cysteine was specified in Mascot as a fixed modification. Deamidated of asparagine and glutamine and oxidation of methionine were specified in Mascot as variable modifications.

Scaffold (version Scaffold_5.0.1, Proteome Software Inc., Portland, OR) was used to validate MS/MS based peptide and protein identifications. Peptide identifications were accepted if they could be established at greater than 90,0% probability by the Peptide Prophet algorithm^70^ with Scaffold delta-mass correction. Protein identifications were accepted if they could be established at greater than 99,0% probability and contained at least 2 identified peptides. Protein probabilities were assigned by the Protein Prophet algorithm.^71^ Proteins that contained similar peptides and could not be differentiated based on MS/MS analysis alone were grouped to satisfy the principles of parsimony. Proteins sharing significant peptide evidence were grouped into clusters.

##### PDX samples protein identification

The resulting MS/MS spectra from PDX samples were processed with MaxQuant using both; UniProt human (Homo sapiens) and mouse (Mus musculus) databases and custom-made tryptic peptide libraries generated from an *in silico* digest of the human and mouse proteomes assuming the digestion enzyme trypsin. iBAQ relative abundance scores were calculated using ‘PaxDb: Protein Abundance Database’.^72^

### Quantification And Statistical Analysis

#### EV and secretome proteomics data

For LC-MS/MS peptide identification was performed using the probability ratio method^73^ and false discovery rate (FDR) was calculated using inverted databases, and the refined method^74^ with an additional filtering for precursor mass tolerance of 10 ppm.^75^ Identified peptides with an FDR equal or lower than 1% FDR were used to quantify the relative abundance of each protein from reporter ion intensities, and statistical analysis of quantitative data were performed using the WSPP statistical model previously described.^76^ Briefly, in this model protein log2-ratios are expressed as standardized variables, i.e., in units of standard deviation according to their estimated variances (Zq values).

### Nascent proteomics data

Analysis of nascent proteomics (OPP-ID and BONCAT) experiments was performed on the normalized spectrum counts obtained from the Scaffold software. The output data was cleaned to exclude any known contaminants mapped to the ‘cRAP’ database and all NA values substituted for 0s. The resulting data frames were then used for different downstream analyses based on the experiment. Any differential expression/enrichment analysis between experimental conditions was carried out using the ‘Differential Enrichment analysis of Proteomics data’ (DEP) R package.^68^

### Statistical analysis

Data shown in figures corresponds to mean ± standard deviation (SD). For statistical analysis, normality and homoscedasticity tests were carried out followed by parametric tests: Log rank (Mantel-Cox) test was used for survival analyses, Analysis of Variance (ANOVA) tests were used for multiple groups and unpaired two-tailed t tests were used for two group comparisons. Significant statistical differences between groups are indicated as: *p < 0.05, **p < 0.01, ***p < 0.001, ****p < 0.0001. All analyses and graphics were carried out with GraphPad Prism 9 software and Microsoft Excel or RStudio.

## Supplementary Material

Supplementary Material

## Data Availability

The MS proteomics data have been deposited at the ProteomeXchange Consortium via the PRIDE partner repository with the dataset identifiers PXD058646 and PXD058648 and are publicly available as of the date of publication. This paper does not report original code. Any additional information required to reanalyze the data reported in this paper is available from the lead contact upon request.

## In brief

Lisi-Vega et al. report that refractory AML is characterized by increased translation in leukemia cells and their niches. BM mesenchymal stromal cells (BMSCs) supply cap-dependent translation machinery to AML cells via extracellular vesicles. Targeting this mechanism with specific inhibitors reveals BMSC-AML translational crosstalk as a potential driver of AML recurrence.

## Highlights

- Increased translation in refractory AML cells and their niches
- BMSCs are translationally reprogrammed in AML
- BMSCs boost AML translation by EV-mediated transfer of translation-related proteins
- BMSC-AML translational crosstalk is key for AML survival after chemotherapy
