# Supplementary material for: Bone marrow mesenchymal stromal cells support translation in refractory acute myeloid leukemia
Source: Cell Rep. Author manuscript; Available in PMC 2025 Mar 2. (PMC7617453; doi:10.1016/j.celrep.2024.115151)
Supplement: Supplementary Material [file EMS203146-supplement-Supplementary_Material.pdf]

Cell Reports, Volume *44*

## Supplemental information

# Bone marrow mesenchymal stromal cells support translation in refractory acute myeloid leukemia

**Livia E. Lisi-Vega, Alice Pievani, María García-Fernández, Dorian Forte, Tim L. Williams, Marta Serafini, and Simón Méndez-Ferrer**

# Figure S1, Related to Figure 1

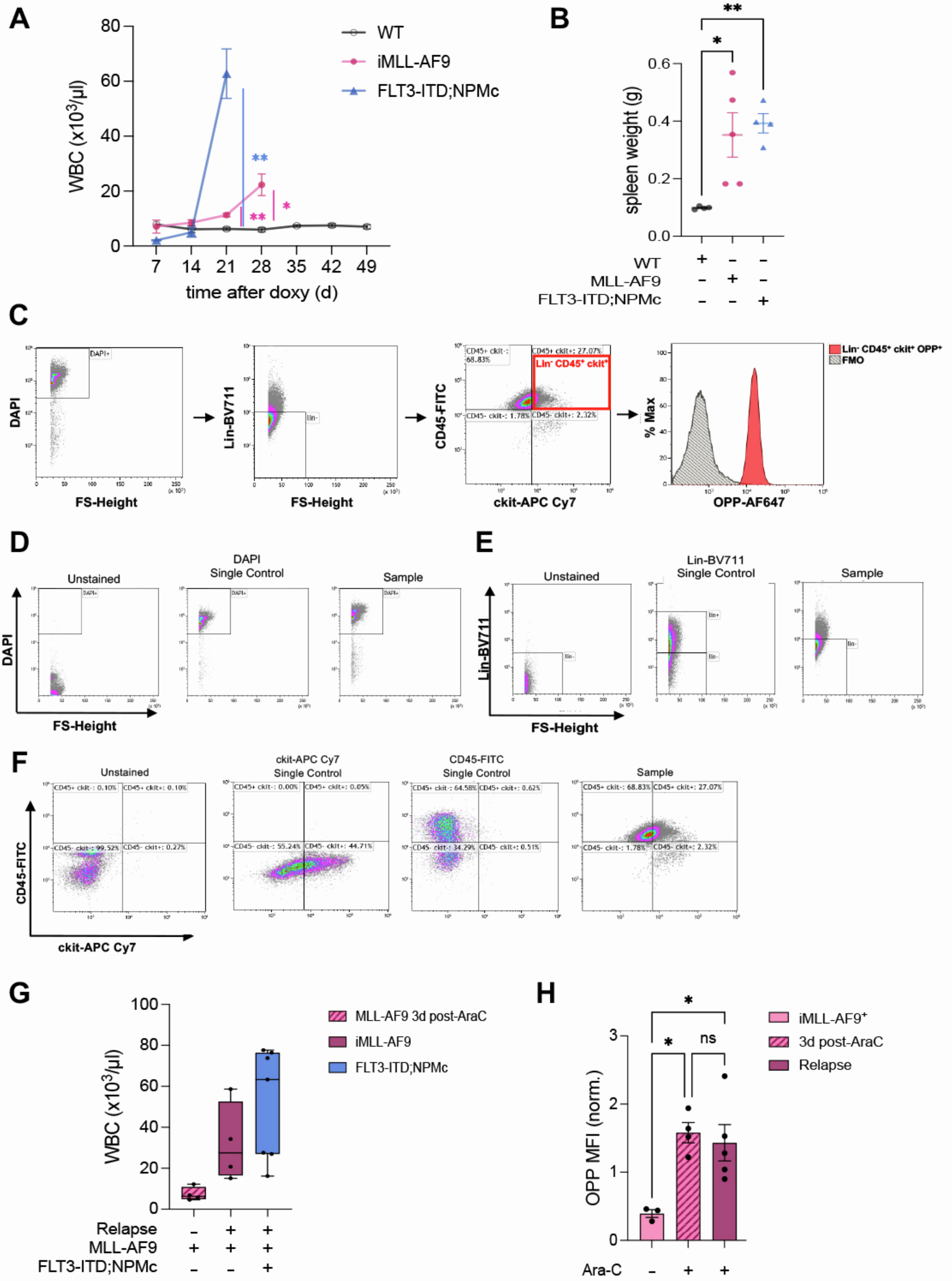

**Figure S1. Related to Figure 1. Increased protein synthesis in AML cells and their niches supports relapse.**

(A-B) Peripheral white blood counts (WBCs) (A) and spleen weights (B) of iMLL-AF9 and FLT3-ITD;NPMc mice compared to control littermates (WT). Dots represent individual mice. Data pooled from 4 independent experiments; *p<0.05; unpaired two-tailed *t* test.

(C) Gating strategy for *in vivo* O-propargyl-puromycin (OPP) experiments assessing the translation levels of $Lin^-$ $CD45^+$ $ckit^+$ (LK) AML cells.

(D-F) Control staining used for flow cytometry comprising unstained and single controls for DAPI, lin-BV711, CD45-FITC and ckit-APC Cy7 antibodies.

(G) Box plots summarising the distribution of white blood cells (WBCs) of relapsed iMLL-AF9 and FLT3-ITD;NPMc mice and iMLL-AF9 3 days after chemotherapy (3 days post-AraC). Lines denote median values for each group.

(H) Comparison of global protein synthesis levels measured by O-propargyl-puromycin (OPP) mean fluorescent intensity (MFI) in MLL-AF9 blasts from iMLL-AF9 mice before chemotherapy treatment, 3 days after chemotherapy (when WBCs are still < $15x10^3/mm^3$) and at relapse, once WBCs exceed $15x10^3/mm^3$. MFI values are normalized to average OPP MFI values of therapy-naïve AML mice.

(B, G-H). Dots represent biological replicates.

(A-B, G-H) Data are mean ± SEM. *p<0.05; **p<0.01. One way ANOVA and pairwise comparisons.

# Figure S2, Related to Figure 1, 2 & 4

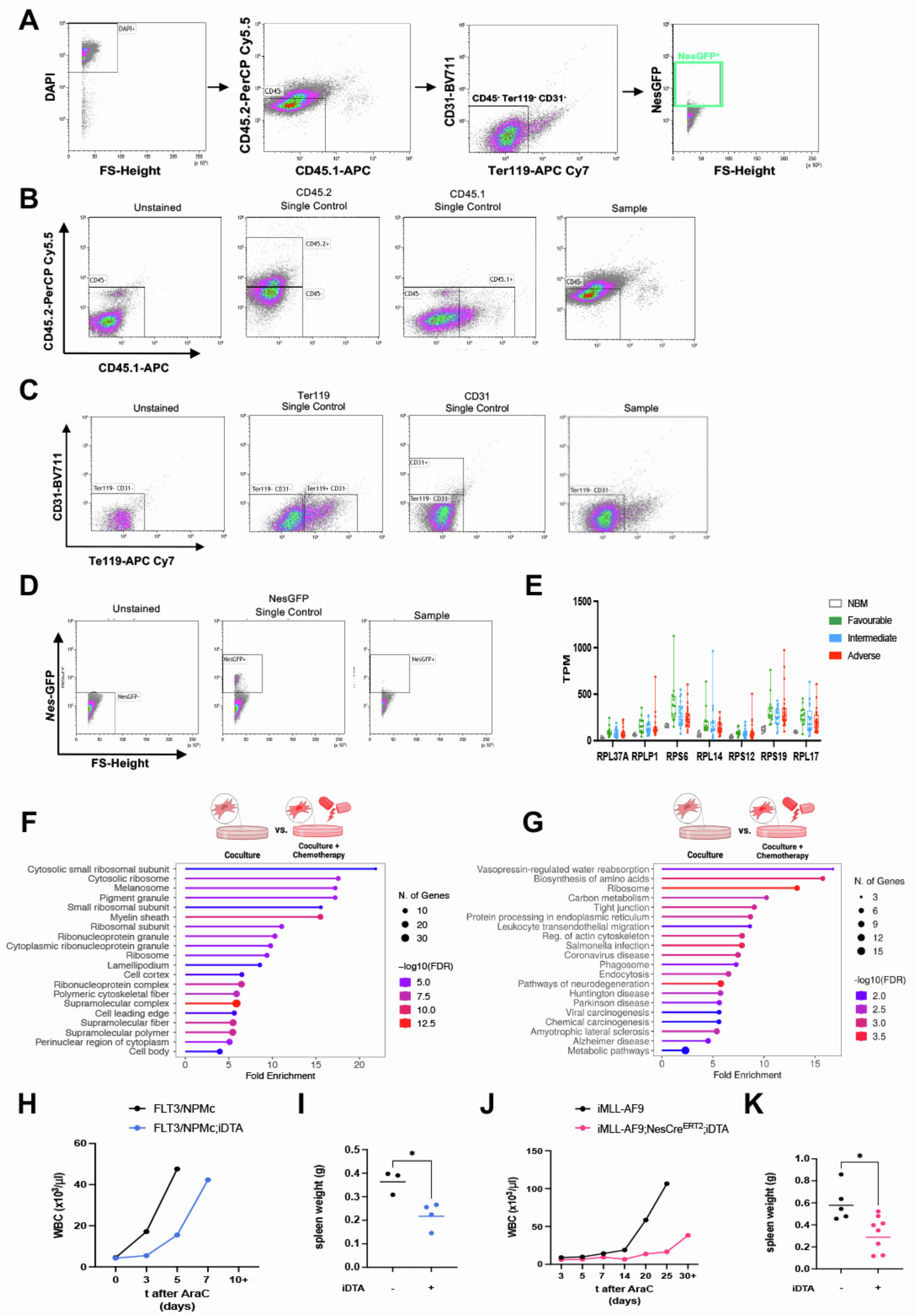

**Figure S2. Related to Figures 1, 2 and 4. Flow cytometry gating of BMSCs and AML models.**

(A) Flow cytometry gating strategy to measure *Nes*-GFP$^+$ BMSCs.

(B-D) Control staining used for flow cytometry comprising unstained and single controls for CD45.2-PErCP Cy5.5, CD45.1-APC, CD31-BV711 and Ter119-APC Cy7 antibodies.

(E) RNA expression levels of selected ribosomal proteins in BMSCs from patients with AML related to distinct ELN2017 genetic risk categories vs. normal BM controls based on bulk RNA-seq data from CD45$^-$CD71$^-$CD235a$^-$CD31$^-$CD271$^+$ AML BMSCs[78].

(F-G) GO cellular component categories (F) and KEGG pathways (G) found enriched in BMSCs cocultured with chemotherapy in comparison to steady state co-culture.

(H-K) Kinetics of AML recurrence (H, J) and spleen weight (I, K) of FLT3-ITD;NPMc mice (H-I) and MLL-AF9 mice (J-K) with (iDTA+) or without (iDTA−) nestin$^+$ cell depletion. Dots represent data from individual mice (average data pooled from 4 independent experiments); *$p<0.05$; unpaired two-tailed t test.

# Figure S3, Related to Figure 4

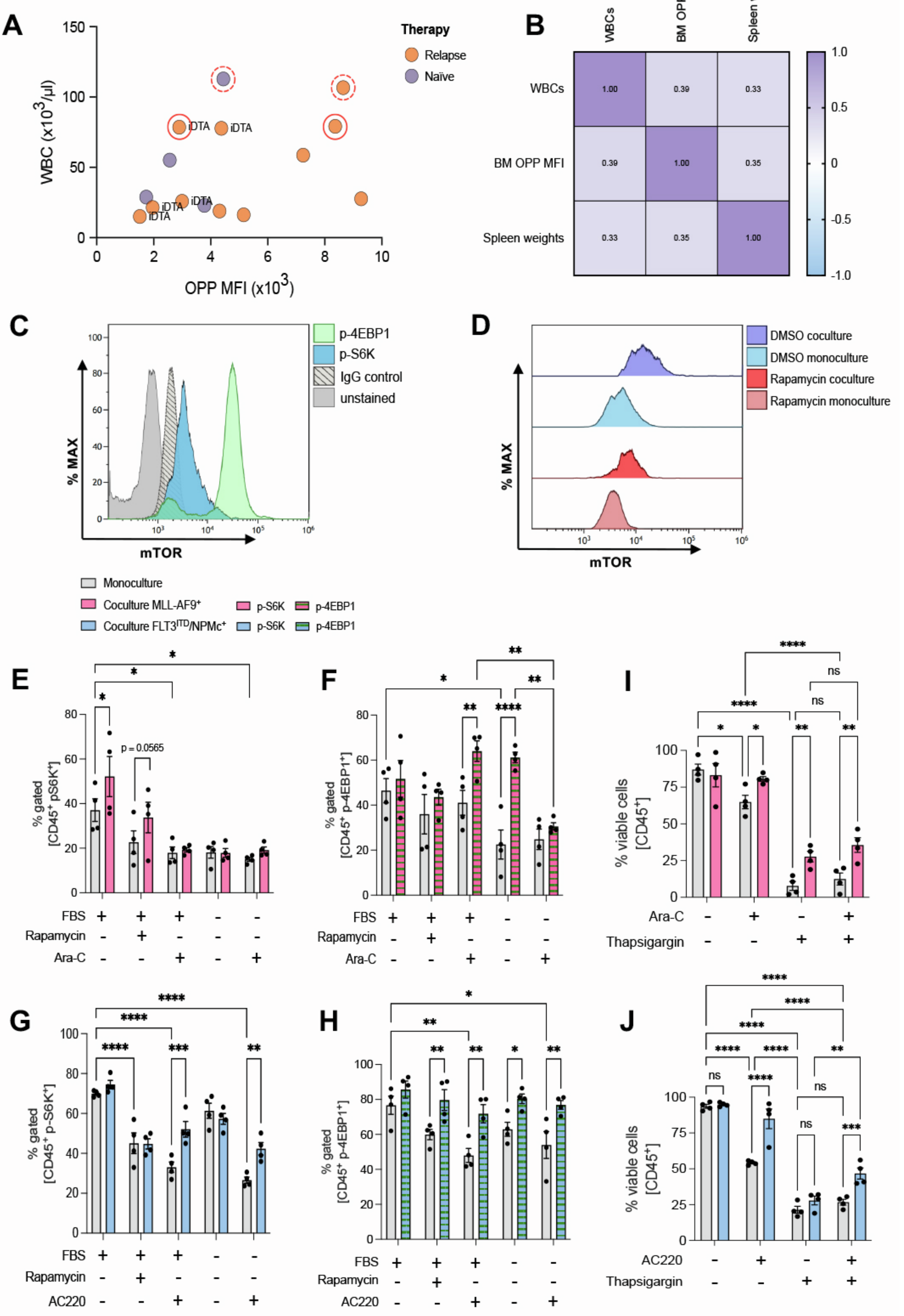

**Figure S3. Related to Figure 4. Nestin⁺ BMSCs support increased protein synthesis at relapse.**

(A) Dot plot indicating the relationship between the different *in vivo* variables such as WBC (Y axis), treatment (naïve vs. relapse) or nestin+ cell depletion (*iDTA*) and global protein synthesis levels as measured by OPP MFI (X axis). Dots represent individual mice. Highlighted in red are those mice with similar disease burden (as measured by WBCs) but different OPP MFI levels based on treatment (naïve vs. relapse, red discontinuous line) or nestin$^+$ cell depletion (control vs. *Nes-Cre*$^{ERT2}$*;iDTA,* red continuous line).

(B) Pearson correlation matrix measuring the strength of the linear relationship between the different *in vivo* variables related to disease burden and global protein synthesis (measured as OPP MFI) in S1D.

(C-D) Representative flow cytometry histograms of phospho-flow controls (C) and of p-S6K-AF488 fluorescent signal (D) in monocultured/cocultured MLL-AF9 AML blasts with/without rapamycin treatment.

(E-F) Percentage of CD45$^+$ p-S6K$^+$ (E) or CD45$^+$ p-4EBP1$^+$ (F) MLL-AF9 blasts cultured alone or in coculture with BMSCs, treated with AraC or with the mTOR inhibitor rapamycin in the presence or absence of FBS for 12h. Dots represent biological replicates (n = 3 independent experiments).

(G-H) Percentage of CD45$^+$ p-S6K$^+$ (G) or CD45$^+$ p-4EBP1$^+$ (H) FLT3-ITD;NPMc blasts cultured alone or in coculture with BMSCs, treated with AraC or with the mTOR inhibitor rapamycin in the presence or absence of FBS for 12h. Dots represent biological replicates (n = 3 independent experiments).

(I-J) Percentage of surviving CD45$^+$ MLL-AF9 (I) or FLT3-ITD;NPMc (J) AML cells cultured alone or in coculture with BMSCs respectively treated with AraC (I) or the FLT3i AC220 (J), with the ER stressor and UPR-inducer thapsigargin, combined therapy (chemo + thapsigargin) or control vehicle for 12h. Dots represent biological replicates (n = 3 independent experiments).

(E-K) Dots represent biological replicates. Data are mean ± SEM. *p < 0.05; **p< 0.01; ***p < 0.001; ****p< 0.0001. One way ANOVA and pairwise comparisons.

# Figure S4, Related to Figure 5

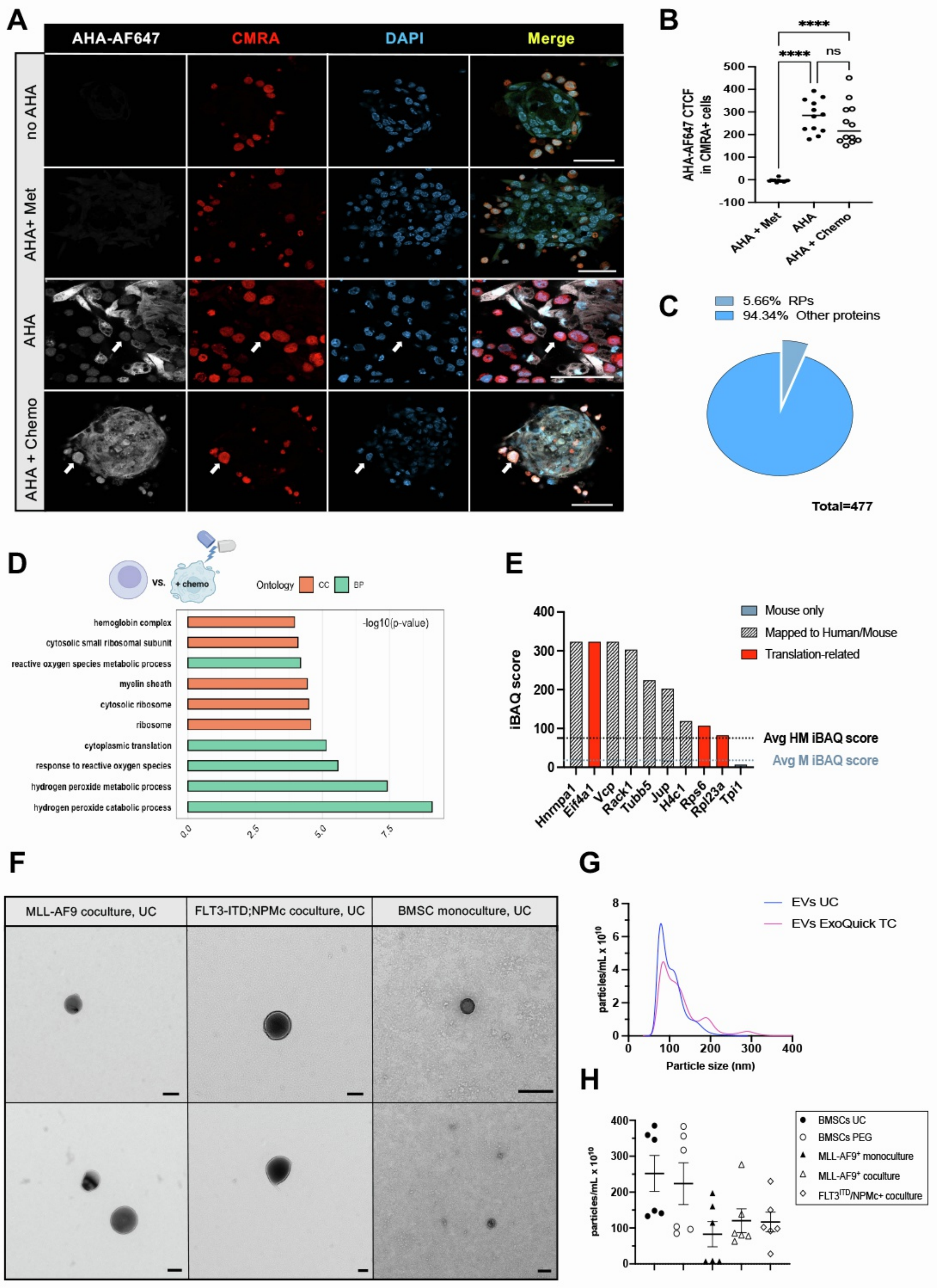

**Figure S4. Related to Figure 5. Translation-related proteins transferred from BMSCs support AML relapse.**

(A) Imaging of nascent protein synthesis through AHA-labelling of BMSCs (grey) before co-culture with CMRA-labelled (red) AML blasts. BMSCs cultured as spheres were pulsed for 5h with AHA only, AHA and Methionine (Met, which competes with AHA for incorporation into nascent proteins and demonstrates specific labelling) or AHA + $H_2O_2$ (to mimic chemotherapy-induced ROS). After the pulse, BMSCs were washed and co-cultured with CMRA-labelled FLT3-ITD;NPMc or MLL-AF9 AML blasts for 24h (AHA = AML blasts cocultured with AHA-labelled BMSCs, AHA + chemo = AML blasts cocultured with $H_2O_2$ pre-treated BMSCs labelled with AHA). Click chemistry was used to fluorescently tag the AHA-labelled proteins with AF647. Unlabeled BMSCs, and BMSCs pulsed with AHA and Met, were used as controls. Arrowheads indicate $CMRA^+$ blasts positive for AHA-AF647. Scale bar, 50mm.

(B) Quantification of AHA-AF647 signal in $CMRA^+$ blasts. Total fluorescence was calculated using the corrected total cell fluorescence method (CTCF). Dots represent data from each coculture well imaged (n=4 independent experiments). Data are means; ****p< 0.0001; One-way ANOVA and pairwise comparisons.

(C) Pie chart representing the frequency of ribosomal proteins vs. total proteins labelled by AHA in monocultured BMSCs.

(D) Gene Ontology (GO) biological process categories over-represented in the list of proteins whose transfer increased from BMSCs in coculture with AML cells after chemotherapy treatment in comparison to steady state coculture.

(E) iBAQ scores from proteins appearing in both FLT3-ITD xenograft and BMSC-AML AHA transfer experiments as microenvironment- and BMSC-derived, respectively. iBAQ scores of translation-related proteins are highlighted in red and average iBAQ scores for mouse and human/mouse mapped proteins are represented by the blue and black discontinuous lines on the Y axis.

(F) Representative transmission electron microscopy images (TEM) of coculture and BMSC-derived EVs isolated by ultracentrifugation (UC) and used for *in vitro* experiments.

(G-H) Size (G) and quantification (particle concentration, H) of EVs isolated from AML monocultures, AML-BMSC cocultures or BMSC monocultures. Data are mean ± SEM.

# Figure S5, Related to Figure 6

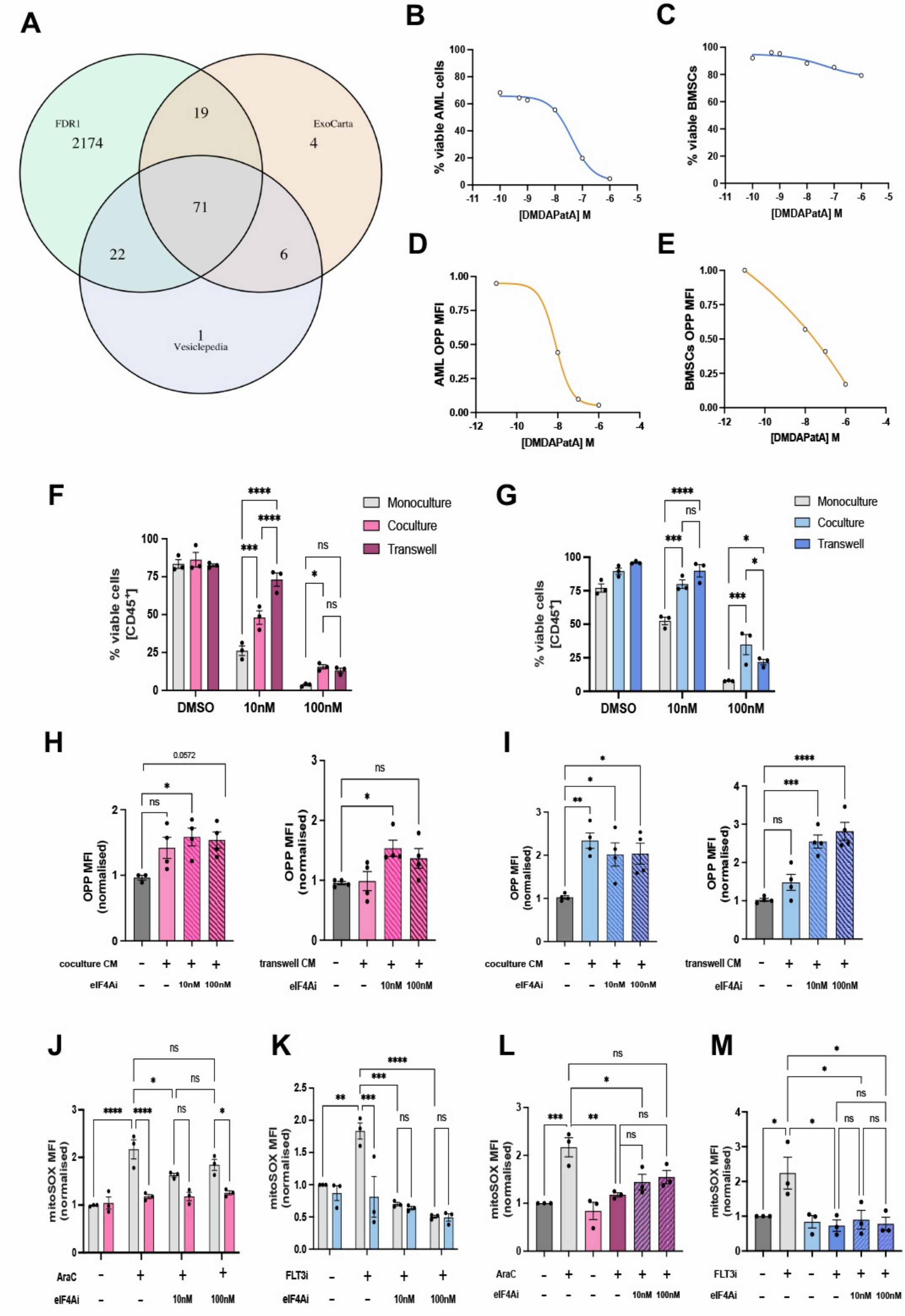

**Figure S5. Related to Figure 6. BMSCs support AML blast translation through eIF4A carried in extracellular vesicles.**

(A) Venn Diagram showing the overlap between proteins identified in the EV proteomics dataset (FDR = 0.1) and the list of top 100 EV marker proteins according to the ExoCarta (90/100 proteins in common) and Vesiclepedia (93/100 proteins in common) databases.

(B-E) Viability (B-C) and O-propargyl-puromycin (OPP) mean fluorescent intensity (MFI) indicative of global translation (D-E) after treatment with different concentrations of the eIFI4A inhibitor DMDAPatA in FLT3-ITD;NPMc blasts (B,D) and BMSCs (C,E) cultured as mesenspheres.

(F-G) Percentage of surviving $CD45^+$ MLL-AF9 (F) or FLT3-ITD;NPMc (G) AML blasts treated 12h with 10/100nM eIF4A inhibitor, or vehicle (DMSO), and maintained 24h in monoculture, or in direct or transwell coculture with BMSCs.

(H-I) Quantification of translation levels (OPP MFI) in (H) MLL-AF9 and (I) FLT3-ITD;NPMc AML cells following the addition of conditioned media (CM) isolated from AML-BMSC coculture or transwell experiments to AML blasts pre-treated with vehicle (DMSO) or eIF4Ai.

(J-K) Mitochondrial ROS levels (measured by mitoSOX$^{TM}$ red) in (J) MLL-AF9 and (K) FLT3-ITD;NPMc AML blasts pre-treated with vehicle or eIF4A inhibitor (eIF4Ai) and maintained alone or in coculture with BMSCs for 24h in the presence or absence of chemotherapy.

(L-M) Mitochondrial ROS levels (measured by mitoSOX$^{TM}$ red) in (L) MLL-AF9 or (M) FLT3-ITD;NPMc AML blasts after 24h of AraC or FLT3 inhibitor (AC220) treatment, respectively, in monoculture or co-culture with BMSCs pre-treated with vehicle or eIF4Ai 12h prior to coculture.

(F-M) Dots represent biological replicates (n = 3 independent experiments). Data are mean ± SEM. *$p < 0.05$; **$p < 0.01$; ***$p < 0.001$; ****$p < 0.001$; (F-I) One way ANOVA and pairwise comparisons. (H-M) Two-way ANOVA followed by Tukey’s multiple comparisons.

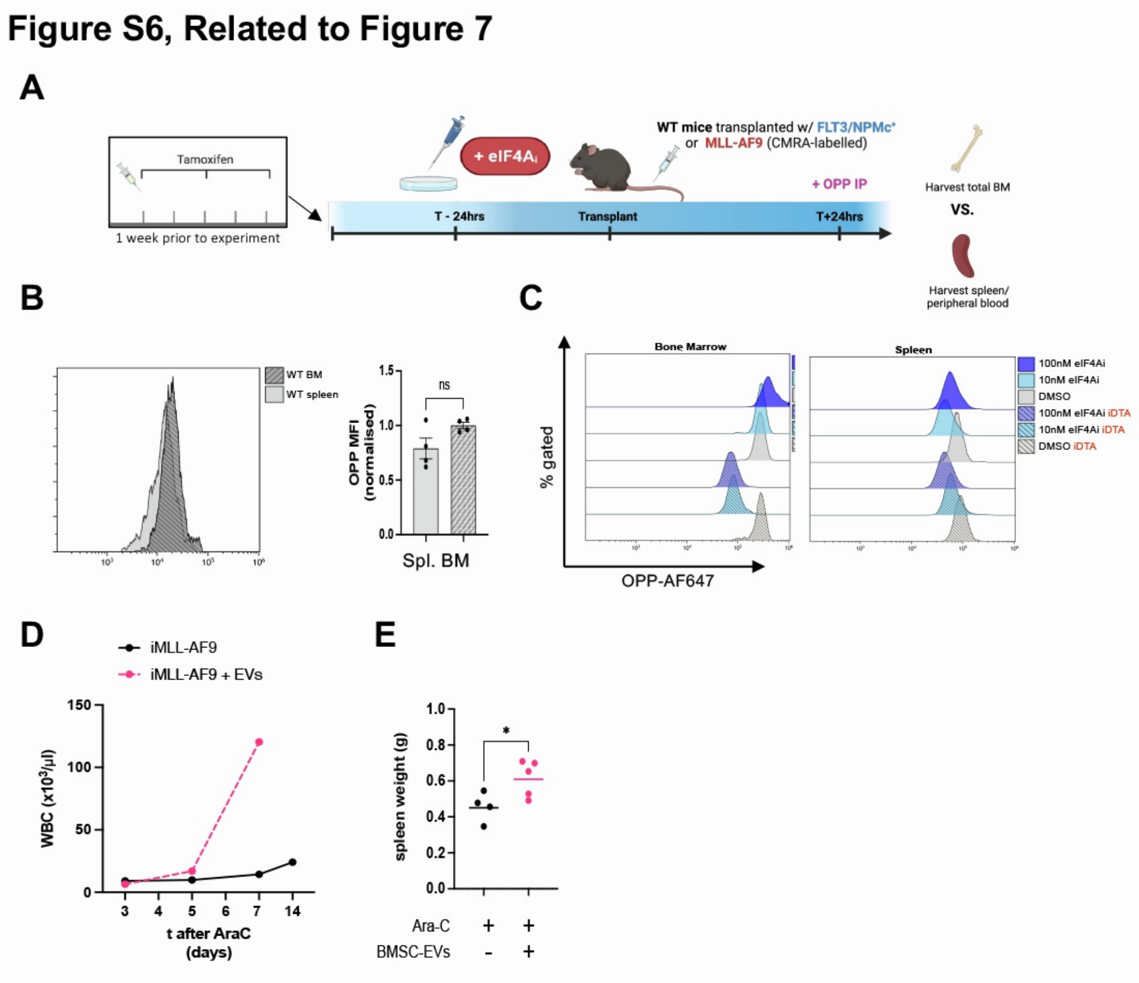


**Figure S6. Related to Figure 7. BMSCs rescue translation inhibition in AML cells *in vivo*.**

(A) Schematic of the adoptive transfer experiment assessing the ability of nestin$^+$ BMSCs to increase AML translation *in vivo*. Briefly, FLT3-ITD;NPMc or MLL-AF9 AML blasts were treated with eIF4Ai or vehicle (DMSO) for 12h, labelled with CellTracker™ Orange CMRA dye and i.v. injected into *Nes-Cre$^{ERT2}$;iDTA* mice (previously treated with tamoxifen to deplete nestin$^+$ BMSCs) or into control littermates. BM and spleen of recipient mice were analyzed 24h later.

(B) Representative histograms of OPP fluorescence and quantification of global translation levels based on OPP MFI in lin$^-$ CD45.2$^+$ ckit$^+$ cells in the BM and spleen of control mice injected with PBS. Dots represent data from individual mice (n = 2 independent experiments). Unpaired two-tailed *t* test.

(C) Representative histograms of OPP fluorescence quantification of global translation levels of CMRA$^+$Lin$^-$CD45.2$^+$cKit$^+$ MLL-AF9 or FLT3-ITD;NPMc blasts previously treated with vehicle or eIF4Ai and harvested 12h after i.v. transplantation from the BM or the spleen of recipient mice with (BM iDTA, spleen iDTA) or without (BM, spleen) nestin$^+$ cell depletion. Two-way ANOVA followed by Tukey's multiple comparisons.

(D-E) Peripheral white blood counts (WBCs) (D) and spleen weights (E) of iMLL-AF9 treated with chemotherapy alone (black) or followed by i.v. injection of BMSC-derived extracellular vesicles (EVs, pink); Dots represent data from individual mice, n = 2 independent experiments, *p<0.05; unpaired two-tailed *t* test.
